# Integrated metabolic modelling reveals cell-type specific epigenetic control points of the macrophage metabolic network

**DOI:** 10.1186/s12864-015-1984-4

**Published:** 2015-10-19

**Authors:** Maria Pires Pacheco, Elisabeth John, Tony Kaoma, Merja Heinäniemi, Nathalie Nicot, Laurent Vallar, Jean-Luc Bueb, Lasse Sinkkonen, Thomas Sauter

**Affiliations:** Life Sciences Research Unit, University of Luxembourg, 162a, Avenue de la Faïencerie, L-1511 Luxembourg, Luxembourg; Luxembourg Centre for Systems Biomedicine, University of Luxembourg, L-4367 Belvaux, Luxembourg; Genomics Research Unit, Luxembourg Institute of Health, L-1526 Luxembourg, Luxembourg; Institute of Biomedicine, School of Medicine, University of Eastern Finland, 70211 Kuopio, Finland

**Keywords:** Metabolic modelling, Macrophage differentiation, High regulatory load, Active enhancer, Regulation of metabolism

## Abstract

**Background:**

The reconstruction of context-specific metabolic models from easily and reliably measurable features such as transcriptomics data will be increasingly important in research and medicine. Current reconstruction methods suffer from high computational effort and arbitrary threshold setting. Moreover, understanding the underlying epigenetic regulation might allow the identification of putative intervention points within metabolic networks. Genes under high regulatory load from multiple enhancers or super-enhancers are known key genes for disease and cell identity. However, their role in regulation of metabolism and their placement within the metabolic networks has not been studied.

**Methods:**

Here we present FASTCORMICS, a fast and robust workflow for the creation of high-quality metabolic models from transcriptomics data. FASTCORMICS is devoid of arbitrary parameter settings and due to its low computational demand allows cross-validation assays. Applying FASTCORMICS, we have generated models for 63 primary human cell types from microarray data, revealing significant differences in their metabolic networks.

**Results:**

To understand the cell type-specific regulation of the alternative metabolic pathways we built multiple models during differentiation of primary human monocytes to macrophages and performed ChIP-Seq experiments for histone H3 K27 acetylation (H3K27ac) to map the active enhancers in macrophages. Focusing on the metabolic genes under high regulatory load from multiple enhancers or super-enhancers, we found these genes to show the most cell type-restricted and abundant expression profiles within their respective pathways. Importantly, the high regulatory load genes are associated to reactions enriched for transport reactions and other pathway entry points, suggesting that they are critical regulatory control points for cell type-specific metabolism.

**Conclusions:**

By integrating metabolic modelling and epigenomic analysis we have identified high regulatory load as a common feature of metabolic genes at pathway entry points such as transporters within the macrophage metabolic network. Analysis of these control points through further integration of metabolic and gene regulatory networks in various contexts could be beneficial in multiple fields from identification of disease intervention strategies to cellular reprogramming.

**Electronic supplementary material:**

The online version of this article (doi:10.1186/s12864-015-1984-4) contains supplementary material, which is available to authorized users.

## Background

Metabolism is a highly regulated dynamic process that involves transport and chemical reactions of thousands of metabolites to fulfill hundreds of metabolic functions. Metabolic dysfunction is a major contributor to many diseases which have become prevalent in human population in the last decades, e.g. cardiovascular diseases [1], neurodegenerative diseases [2] and cancer [3] amongst many others. Alternative pathways and branches are continuously activated or shut down to maximize metabolic efficiency in a specific context [4], resulting in disease and patient-specific alterations.

Metabolism is regulated at multiple-levels with abundance and expression of the metabolic enzymes being one of the most decisive mechanisms. Gene expression control has to integrate multiple signals both at transcriptional and post-transcriptional levels. At the epigenetic level the availability of various transcription factor (TF) binding sites through chromatin decondensation at context-specific enhancers is regulated by the interplay of TFs and post-translational histone modifications deposited by the recruited co-activators [5]. Enhancers adhere to unique chromatin states defined by features such as deposition of histone variants, presence of co-activators and monomethylation of histone H3 at lysine 4 (H3K4me1) [6]. More recently, acetylation of histone H3 at lysine 27 (H3K27ac) was described to specifically mark active enhancers engaged in regulation of RNA polymerase activity through chromatin looping [7, 8]. Recent work on genome-wide analysis of active enhancers has revealed that important genes determining cellular identity, such as TFs, are often controlled by large and strong clusters of multiple enhancers called super-enhancers or stretch-enhancers that are active in a cell type-specific manner [9–11]. Moreover, these enhancer clusters usually reside in insulated chromatin loops or domains and often overlap with so called TF hotspots, suggesting that their target genes are under high regulatory load from multiple TFs and enhancers, integrating numerous different signals to promote proper cellular phenotype, including the appropriate metabolic network [12, 13]. However, the role of high regulatory load genes in the metabolic networks has not been studied previously.

Metabolic networks are highly complex and can hardly be understood without using mathematical representations. The most comprehensive descriptions of metabolism are genome-scale reconstructions (GENREs). There are several human reconstructions available, like Recon 1 and Recon 2 [14, 15] or the Edinburgh Human Metabolic Network [16]. Alongside with these reconstructions extensive reaction databases were developed, like HMR [17, 18] or HumanCyc [19, 20], which collect additional information to refine the available models. Mathematical models derived from GENREs were successfully used to understand how perturbations in the metabolism lead to severe pathologies [18, 21, 22].

GENREs are usually generic representations of a cell or organism comprising all reactions that can potentially become active regardless of the specific environment and cell type. Therefore they do not cover the fact that the set of expressed genes and thereby the set of active reactions vary significantly in function of the cellular context. The generation of context-specific models that include only pathways predicted to be active in the given context is highly desirable and has lead to the development of various algorithms like GIMME [23], IMAT [24], MADE [25], mCADRE [26], INIT [17] or MBA [27], that use omics data for building of context-specific model. While allowing the generation of models with higher predictive power than the GENREs from which they were derived from [23, 27], these algorithms suffer from high computational demands due to the application of mixed integer linear programming, and/or the required setting of one or several expression thresholds.

Recently we proposed an LP-based algorithm for the fast reconstruction of compact context-specific metabolic networks (FASTCORE) that allowed decreasing the reconstruction time of context-specific networks to the order of seconds, using as input a GENRE and a set of core reactions being active in the context of interest [28]. FASTCORE identifies a close to minimal set of non-core reactions from the input model, to be added to the core set in order to obtain a consistent model.

To adapt FASTCORE for the direct integration of microarray data, we propose here a new workflow: FASTCORMICS pre-processes microarray data with the discretization tool Barcode [29, 30], is devoid of arbitrary parameter settings and has a low computational demand with overall context-specific model building times in the order of a few minutes. We use FASTCORMICS to generate multiple metabolic models across tens of primary cell types and analyze the cell type-specific usage of the alternative branches in metabolic networks. To address the question of epigenetic regulation of metabolism in different cell types we performed genome-wide mapping of active enhancers in primary human macrophages and integrated these data with metabolic models of monocyte-to-macrophage differentiation to expose the metabolic genes under high regulatory load by multiple enhancers. We show that high regulatory load genes have a cell type-selective expression profile within any metabolic pathway and a specific positioning of many of these genes at transport or entry point reactions of pathways.

## Results

### Analysis of cell type-specific metabolic networks of primary human cells

In order to adapt FASTCORE for the integration of transcriptomics data from microarrays, we developed a new workflow named FASTCORMICS (Additional file 1: Figure S1), requiring as inputs microarray data, which are first pre-processed with the discretization tool Barcode [30], and a GENRE of the organism of interest. Like FASTCORE, FASTCORMICS is devoid of arbitrary parameter settings and has a low computational demand with overall building times in the order of a few minutes.

To validate FASTCORMICS, we first performed an essentiality assay on two generic cancer models based on Recon 1 and Recon 2 and existing microarray expression data from 59 cancer cell lines [31, 32] (for full description, please see Additional file 1). Comparison to a ranked gene list based on an shRNA essentiality screen in several different cancer cell lines [33] shows the significant predictive power of the FASTCORMICS models (Additional file 1: Table S1). Benchmarking against similar algorithms shows that FASTCORMICS clearly outperforms competitors in speed, while predicting the highest number of essential genes and achieving best significance levels among other algorithms (for results and medium composition, see Additional file 1: Table S1 and Additional file 2: Table S2, respectively). A hypergeometric test also showed that the neoplasia-associated genes retrieved from the DisGeNet database [34] are over-represented in the essential genes of both FASTCORMICS models (Additional file 1: Table S3). Finally, predicted lactate secretion rates based on cancer cell line specific reconstructions showed a good correlation with measured rates indicating the capability of FASTCORMICS to also generate context specific reconstructions (Additional file 1: Figure S2).

**Fig. 1:**
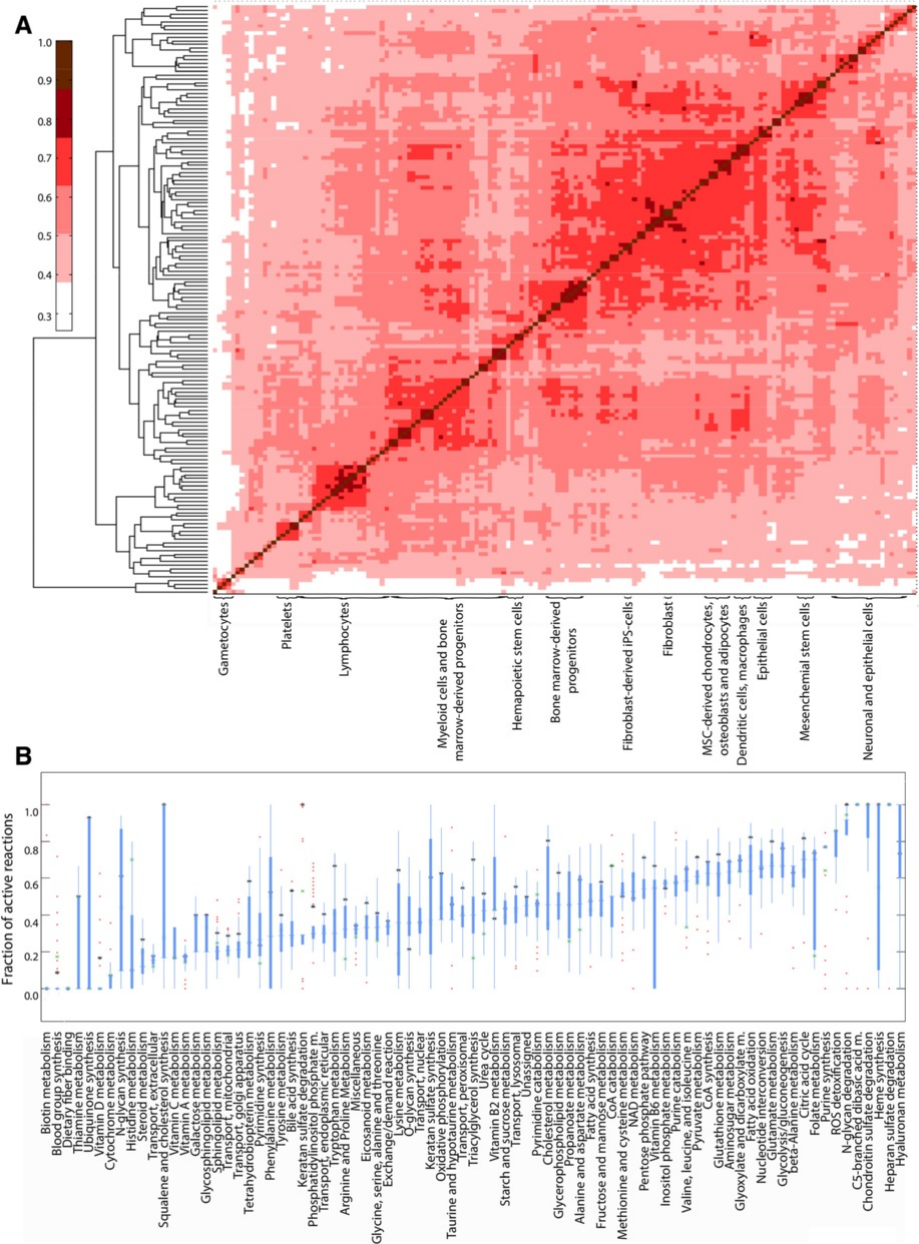
Identification of cell type-specific metabolic pathways in primary human cells. **a** 156 metabolic models based on an equal number of microarrays and corresponding to 63 primary human cell types were built using the FASTCORMICS workflow and microarray collection from Primary Cell Atlas (GSE49910). The level of similarity between the different model pairs was determined via the Jaccard index. The Jaccard index matrix was then clustered in function of the similarity level. **b** For each pathway, the level of activity, given as percentage of reactions of the consistent version of Recon 2 (5317 reactions) that are present in each context-specific model was computed. The distribution of the activity levels of each pathway across the 156 models are shown as box plots and sorted according to the median value across the pathways. Pathways that contain less than 4 reactions were not included. Mean percentage of active reactions across macrophage and monocyte samples are depicted by black asterisk (*) and green cross (x), respectively

In order to identify cell type-specific differences in the usage of the human metabolic network and to further validate the FASTCORMICS workflow, we generated context-specific metabolic models based on Recon 2 for different cell types across most human lineages. From an existing collection of 745 microarrays [35], we selected a subset of 156 microarrays (Additional file 3: Table S4), corresponding to 63 primary human cell types at their resting states, and took advantage of the low computational demands of FASTCORMICS to generate a model for each microarray. All reconstructed models are available in SBML format (Additional file 4). Interestingly, clustering the different models according to their active reactions allowed clear separation between the cell types largely along their developmental origin or cellular function, suggesting significant differences in the metabolism across cell types (Fig. 1a). The most unique metabolism was predicted for the gametocytes, oocytes and spermatocytes, which at lowest showed only around 30 % similarity to other cell types. Some of the largest clusters were formed by the different blood cells that clustered together with their progenitors as well as CD34^+^ hematopoietic stem cells, suggesting many shared features in their metabolism.

To investigate how much the different pathways contribute to the differential metabolism between the cell types, and what are the most unique pathways in different cell types, we looked into the activity state of all reactions according to the pathways they belong to. Figure 1b lists all the Recon 2 pathways consisting of more than one reaction, ordered by their combined median activity in all analyzed cell types with the first pathways (from left to right) showing no activity in almost none of the analyzed cell types and the last pathways being fully active in almost all cell types. The distribution of these values indicates the variation in the number of active reactions for each pathway between cell types and, for example, the usage of additional or alternative branches of the pathways. By focusing on the most deviant values of any pathway one can identify the cell types that show very high or very low number of active reactions for that pathway compared to other cell types, and can thereby identify the cell type-specific branches of those pathways.

Altogether, as expected, the different cell types exhibit differential usage of their metabolic pathways, ranging from ubiquitous to cell-type specific. This variation can be captured by FASTCORMICS and allows clustering of the cell types according to their functions and developmental origins.

### Metabolic modelling of primary human monocyte-to-macrophage differentiation

**Fig. 2:**
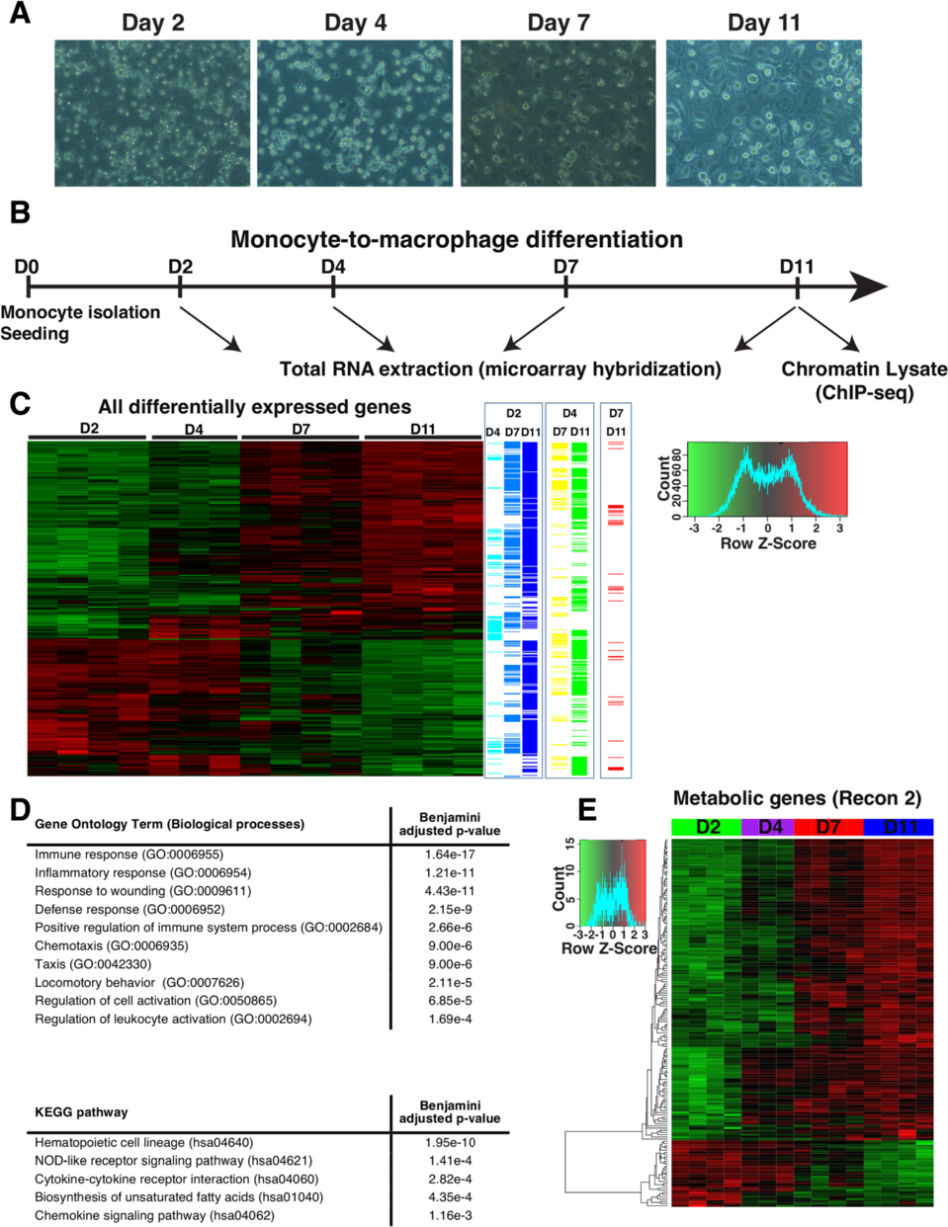
Transcriptomic profiling of primary human monocyte-to-macrophage differentiation. **a**-**b** Primary human monocytes isolated from donated blood samples were differentiated into monocyte-derived macrophages *in vitro*, and microarrays were performed with total RNA extracted on time points day 2, day 4, day 7 and day 11. In addition chromatin was isolated for Chip-seq experiments from day 11 macrophages. **c** Relative expression levels of differentially expressed genes during monocyte-to-macrophage differentiation selected with a FDR cut-off of 0.05 and absolute fold change greater or equal to 2 were clustered and represented as a heatmap. Genes with a positive Z-score are represented in red and negative in green. On the right of the heatmap, the time points where the differentially expressed genes show significant changes are indicated for a comparison between D2 and the remaining time points in different shades of the blue, between D4 and D7 or D11 in yellow or green and between D7 and D11 in red, indicating that most significant expression changes occur already at early time points. **d**. A Gene ontology analysis for enriched biological processes and KEGG pathways was performed on the differentially expressed genes using DAVID [73]. The top ten gene ontology terms for the biological processes and the top five KEGG pathways are listed. **e** Relative expression levels of Recon 2 genes with differential expression (absolute fold change greater or equal to 2 and FDR < 0.05) during monocyte-to-macrophage differentiation are represented as a heatmap as in panel **c**

One of the cell types with particularly high proportion of active reactions compared to other cell types across many metabolic pathways are macrophages. This is true when comparing to the median of all cell types as well as when comparing to the immediate precursor cells, the monocytes (Fig. 1b, Additional file 1: Figure S3). To gain more detailed understanding of the differential usage and regulation of the metabolic pathways in macrophages, we chose to generate our own expression data with sampling at multiple time points during differentiation of primary human monocytes to macrophages as well as regulatory data from macrophages by mapping active enhancer regions (Fig. 2a). This was done in multiple biological replicates to stringently focus on regulatory regions that are active in most healthy individuals (please see next chapter for details).

For the expression profiling we chose to isolate primary human monocytes from blood samples of four healthy donors and to differentiate those to adherent mature macrophages over a time course of 11 days (Fig. 2a). Total RNA was collected at four time points, 2, 4, 7 and 11 days after isolation, and used for gene expression profiling by microarrays (Fig. 2b). Time points before 2 days were not considered as the cells at these early stages are affected by the stress from the collection and isolation. During the differentiation (comparing day 11 to day 2), a total of 882 genes were significantly up-regulated (FDR < 0.05, log_2_ fold change ≥ 1) while 519 were down-regulated (Fig. 2c, Additional file 5: Table S5). Most expression changes occurred already early in the differentiation and were not too dynamic, as most genes that changed significantly during the differentiation (day 4 or day 7), also remained differentially expressed in day 11 macrophages (Fig. 2c). Gene Ontology (GO) and KEGG Pathway analysis of the differentially expressed genes revealed enrichment for many categories and pathways related to macrophage function, suggesting the differentiation had been successful (Fig. 2d). The differentially expressed genes included also 57 TFs. Among the highest expressed TFs in macrophages we found CEBP-family factors (CEBPB, CEBPA, CEBPD and CEBPG), EGR2, SPI1 (also known as PU.1), SREBF2, and FLI1, most of which are known regulators of macrophage differentiation and phenotype [36–39]. RREB1 was the only factor among the 20 highest expressed TFs for which we did not find any previously described role in macrophages. Finally, 164 metabolic genes became differentially expressed with a log_2_ fold change ≥1 (FDR < 0.05) during the differentiation, most of which were up-regulated (Fig. 2e).

**Fig. 3:**
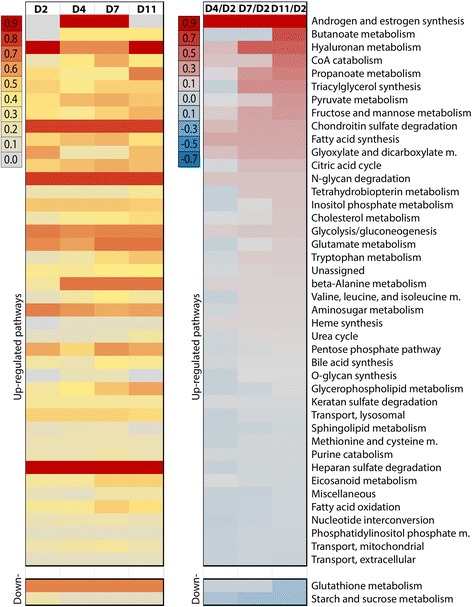
Monocyte-to-macrophage differentiation is accompanied by activation of alternative metabolic branches and increased activity of already active pathways. For each pathway, the level of activity (percentage of reactions in the input model that are present in the context-specific model) was computed for each time point (left panel). Each column represents the model built by the FASTCORMICS workflow for the given time point whereas each line stands for a different pathway. The fraction of active reactions ranges from 0 to 1 and is represented in shades of gray for low, yellow for intermediate and red for high number of active reactions per pathway. Additionally, (right panel) the significantly differentially expressed genes (FDR <0.05 and absolute log_2_ fold change > 1) were mapped to the models via the GPR rules. The percentage of up-regulated reactions in a pathway was computed after summing up the significantly up-regulated reactions. The number of significantly down-regulated reactions was then removed from this sum and the total was then normalized by the number of reactions in the pathway. The fraction of reactions associated with differentially expressed genes ranges between −0.7 for down-regulated pathways in blue and 0.9 for unregulated pathways in red. Only pathways that show a differential expression over time are represented

The microarray data was used as an input for FASTCORMICS to generate four metabolic models that correspond to each tested time point of macrophage differentiation (Fig. 3). All reconstructed models are available in SBML format (Additional file 4). Out of 5317 reactions in the consistent Recon 2 (version 3), 660 reactions were predicted to be active in each time point of macrophage differentiation (Additional file 1: Table S6). The complete size of the day 2 monocyte model was 978 active reactions (corresponding to 64 pathways), which increased to 1149 active reactions (67 pathways) in day 11 macrophages, suggesting that many inactive alternative branches become active during differentiation. Many of the newly activated reactions were turned on already early on day 4 of differentiation with most of the remaining reactions becoming active by day 7. The number of reactions that became inactive in macrophages is smaller with only one pathway decreasing its overall number of active reactions.

Among the pathways with highest relative number of active reactions in macrophages were several fairly ubiquitously active pathways such as hyaluronan metabolism, chondroitin sulfate degradation, and N-glycan degradation (Figs. 1 and 3). However, most of these, as well as many other pathways with steady overall number of active reactions (such as triacylglycerol synthesis and cholesterol metabolism) still showed a significant increase in the expression of the genes corresponding to their active reactions, suggesting a further increased flux for these pathways in macrophages (Fig. 3). The total of 42 subsystems that showed an increase in the expression of genes controlling them, are listed in Fig. 3, together with the 2 subsystems showing decreased activity. Cross-validation based determination of confidence levels of the included model reactions (Additional file 1: Table S7) show high or moderate confidence for approx. 80 % of the reactions, indicating that only approx. 20 % of the reactions did not have expression based support, i.e. were added by FASTCORMICS to generate a consistent network model. And approx. 88 % of the excluded reactions had multiple evidences (reactions with low expression) for not being included.

Next we aimed to find out which subsystems are particularly active in macrophages when compared to other cell types, including monocytes, and therefore possibly under macrophage-specific regulation. Since our own data were generated with more recent Affymetrix arrays (Human Gene 1.0 ST platform) where limited possibilities for comparisons to public data exist, we focused here also on the macrophage samples from the Primary Cell Atlas [35]. Results are depicted in Fig. 1b and Additional file 1: Figure S3. Among the interesting subsystems, for example, more than 60 % of reactions in tryptophan metabolism are predicted active in macrophages while the median value across cell types is 30 %. This is consistent with the models of monocyte-to-macrophage differentiation from our own data, which suggest over 3-fold increase in active tryptophan metabolism reactions over the time course (Fig. 3). Similarly, approximately 80 % of reactions in cholesterol metabolism are predicted active in macrophages, compared to a median of 45 %. Also here there is a comparable 2-fold increase in active reactions from day 2 monocytes to day 11 macrophages in the models based on our own microarrays. Consistently with increased cholesterol metabolism, also bile acid synthesis, a major cholesterol catabolism pathway, is predicted to have more active reaction in macrophages (>50 %) than the median across other cell types (29 %), Other interesting pathways with particularly high numbers of reactions in macrophages include triacylglycerol synthesis and valine, leucine and isoleucine metabolism, both of which show further increase in expression during differentiation from monocytes to macrophages. Overall these results suggest that some alternative branches of the above-mentioned pathways could be under cell type-specific regulation in macrophages.

Taken together, time course analysis of metabolic models during macrophage differentiation predicts changed activities for hundreds of reactions, many of which occur already at early time points and, in contrast to what could be assumed from transcriptome-wide expression level changes, consist largely of increased reaction activities, especially in alternative branches of already active pathways.

### Identification of metabolic genes under high regulatory load in macrophages

Recent work has shown that active enhancers directly involved in transcriptional activation via chromatin looping are marked by specific chromatin modifications such as acetylation of lysine 27 of histone H3 (H3K27ac) [7, 8]. Moreover, we and others have shown that genes under high regulatory load from multiple TFs are often disease-associated and acting as cell type-specific key regulators of cellular identity [11, 40, 41]. Importantly, these genes are marked by a high number of strong enhancers, collectively also called super-enhancers or stretch-enhancers [9, 10], allowing their identification using epigenomic mapping of active enhancers.

**Fig. 4:**
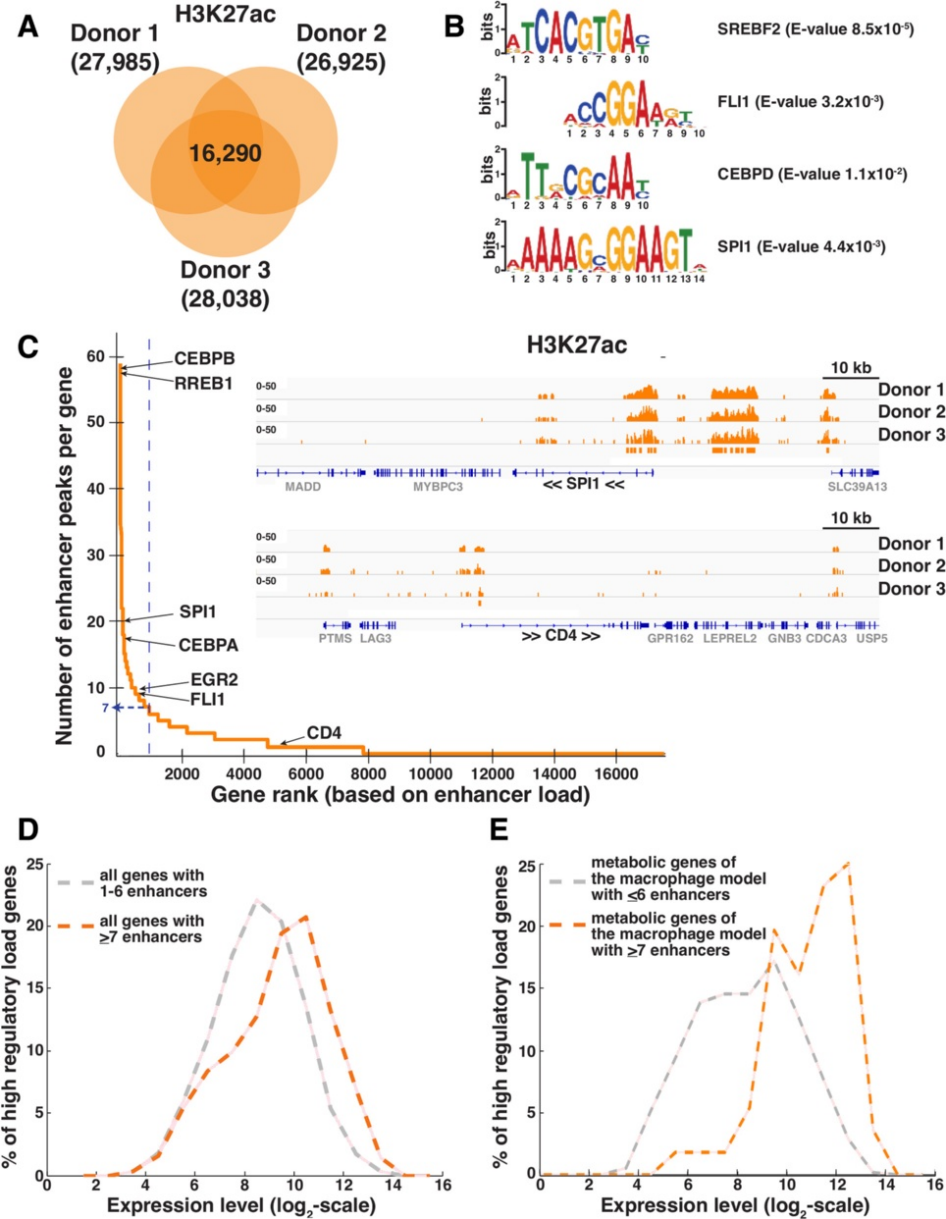
Identification of high-regulatory load genes in human macrophages. **a** Active enhancer regions were identified via chromatin immunoprecipitation coupled to high throughput sequencing (ChIP-Seq) with an antibody against H3K27ac using chromatin from monocyte-derived day 11 macrophages from 3 anonymous donors. Enhancer regions were considered reproducibly detectable when their genomic coordinates overlapped by at least one nucleotide in all biological replicates. **b** Selected enriched sequence motifs located within the identified active enhancer regions associated to upregulated genes in macrophages and corresponding to known transcription factor binding sites are shown. See full list in Additional file 1: Figure S4. **c** Genes associated with at least one active enhancer region were ranked in function of the number of active enhancer regions. A threshold (blue line) corresponding to the top 10 % and at least 7 active enhancer regions was set to segregate between high regulatory load genes and the remaining expressed genes (please see Discussion for details on the threshold selection). 105 kb genomic regions surrounding SPI1 and CD4 loci, mapped reads indicating H3K27ac enrichment from the three donor samples, and called reproducible peaks are shown as examples of high regulatory load and low regulatory load genes, respectively. **d** The distribution of the expression levels of the high regulatory load genes was compared to genes that have a number of enhancers below the threshold of seven enhancers but that are associated to at least one enhancer (KS-test, *p*-value = 4.63e-38). **e** The enhancer load of the metabolic genes present in the consistent version of Recon2 was determined and then manually curated to minimize false peaks-to-gene assignments allowing identification of 74 high-regulatory load genes (≥7 enhancers), 55 of which mapped to the macrophage model. The distribution of expression levels of these metabolic high regulatory load genes was compared to the distribution of expression of the remaining metabolic genes of the macrophage model (KS-test, *p*-value = 1.8537e-11)

In order to identify metabolic genes under high regulatory load in macrophages, we performed chromatin immunoprecipitation coupled to high throughput sequencing (ChIP-Seq) with an antibody against H3K27ac in primary human macrophages derived from additional three donors different on top of the donors used for the microarray analysis. Analysis of the obtained sequencing data identified approximately 27,000-28,000 active enhancer regions in macrophages, depending on the sample, with 16,290 regions detected in all three samples (Fig. 4a). The reproducibly identified enhancers in proximity of induced genes correspond to binding sites of known macrophage TFs such as SREBF2, FLI1, CEBP-family and SPI1, as suggested by the *de novo* motif analysis of the underlying sequences for enriched motifs (Fig. 4b, see Additional file 1: Figure S4 for the complete list).

When assigning the enhancer regions to their putative target genes (see Materials and Methods; Generation of enhancer-to-gene associations), we observed that almost 8000 genes were associated with at least one active enhancer in macrophages, despite our stringent selection (Fig. 4c). Ranking the genes according to their regulatory load (number of associated enhancers) revealed that the number of enhancers per gene ranged from 1 up to 59 with only the top 10 % of the associated genes having 7 or more enhancers. Among these top genes were numerous TFs, many of which were already identified as highly expressed and enriched for their binding site motifs, including CEBP-family members, SPI1, and FLI1. As an example of a high regulatory load gene, the genomic locus of SPI1 – the well-known pioneering factor and key regulator of macrophage differentiation – with two large clusters of multiple enhancers, is depicted in Fig. 4c. In contrast another abundantly expressed macrophage gene, CD4, is using only one intragenic enhancer region. Interestingly, RREB1, which we had previously noticed among highly expressed TFs in our microarray data, but for which no role in macrophages has been described, was the gene with third highest enhancer load of all genes in our experiments, suggesting that RREB1 might play an important role in macrophages or their differentiation. Finally, analysis of the expression levels of the top genes with ≥ 7 associated enhancers confirmed them to be on average significantly higher expressed than the genes with fewer enhancers (KS-test, *p*-value = 4.63e-38; Fig. 4d).

Next we focused on the identification of the metabolic genes under high regulatory load. In total there are 689 metabolic genes expressed in the macrophages that are consistent with our metabolic model and 55 of them belong to genes under high regulatory load of 7 or more enhancers in our data set (based on manual curation of the enhancer to gene association, see Materials and Methods). Importantly, the expression of the metabolic genes under high regulatory load is even more shifted towards high expression levels when compared with other expressed metabolic genes (KS-test, *p*-value = 1.8537e-11; Fig. 4e).

In summary, we reproducibly identified over 16,000 active enhancers in primary human macrophages, a large proportion of which could be associated to the top 10 % of genes with high regulatory load. These genes are expressed at high levels and include many of the known key regulators of macrophage phenotype as well as 55 metabolic genes.

### Genes under high regulatory load control macrophage-specific control points of metabolic pathways

**Fig. 5:**
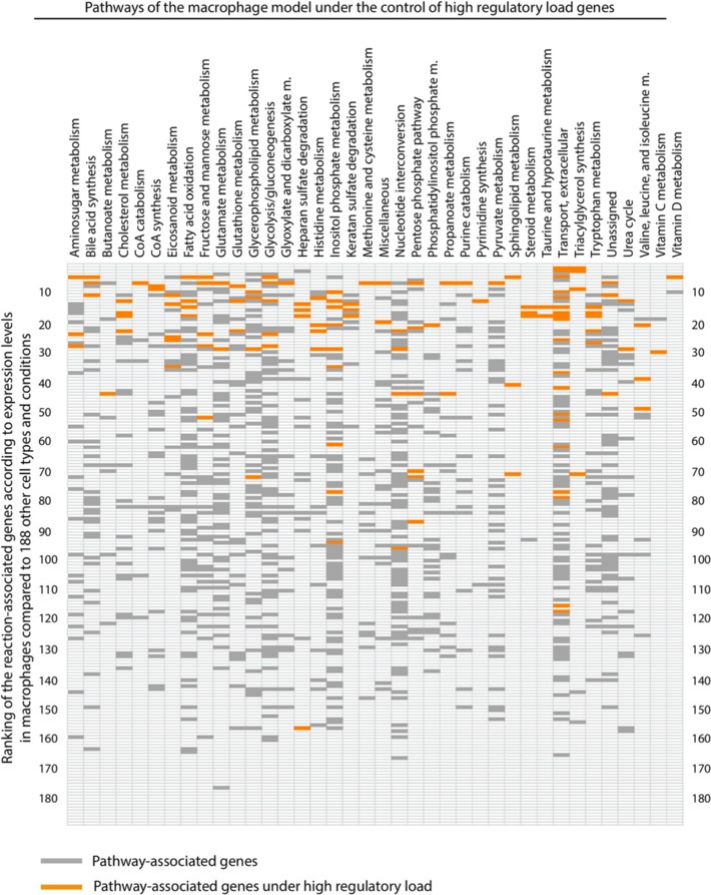
High-regulatory load genes show macrophage specific expression and are the highest expressed genes in their respective pathways. The normalized expression values of the 745 arrays of Primary Cells Atlas were downloaded from the Gene Expression Omnibus repository (GSE49910). The 745 arrays are subdivided in 188 separate cellular contexts. For each reactions-related gene of a pathway, the normalized expression value was retrieved and for each gene, the 188 conditions were ranked from the highest expressed to the lowest expression level with the ImpAvRank function from [74]. For each pathway, the genes are plotted in function of the rank of the monocyte-derived macrophages among the 188 conditions. Each rank position is represented as a box along the y-axis. High-regulatory load genes are mapped along this axis in function of their rank and depicted in orange, whereas the remaining genes in the pathway are depicted in dark gray

Given that genes with high regulatory load are important for the cell identity and often expressed in a cell type-specific manner, we decided to analyze the expression levels of the macrophage metabolic model genes across numerous different cell types. To this end, we again used the microarray data collection from Mabbott et al. [35], this time taking advantage of all 756 arrays corresponding to a total of 188 different cell types and conditions, and analyzed the expression level of each metabolic gene across the 188 conditions and ranked it according to its average level in the monocyte-derived-macrophage samples contained in the data set. Figure 5 depicts these ranks for all genes of the macrophage-specific metabolic model that belong to a subsystem containing at least one high regulatory load gene. Analysis of the distribution of the expression ranks along the cell types and subsystems reveals that; 1) the genes under high regulatory load (marked in orange) show an overall shift towards the upper ranks of macrophage metabolic genes, arguing they are generally expressed in a macrophage-specific manner, and 2) they are the more selectively expressed genes within each metabolic subsystem (Fig. 5). At the same time the other genes contained in the macrophage model show an even distribution across the ranks, suggesting a more ubiquitous expression between cell types.

**Fig. 6:**
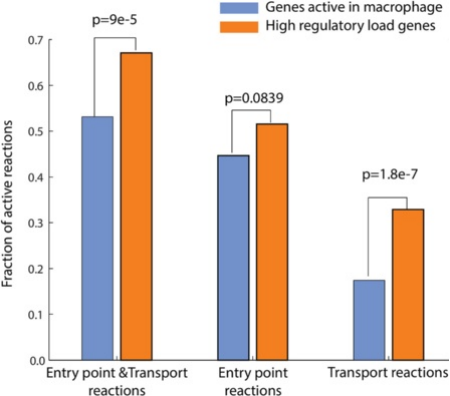
High regulatory load genes control transport and entry point reactions in macrophages. The enrichment of transport reactions and other entry point reactions under high-regulatory load among the gene-regulated reactions of the macrophage model was computed using hypergeometric test. An entry point is defined as the first reaction of a pathway change when considering the flux direction. In addition, the transport reactions and entry point reactions were tested separately to estimate their contributions to the observed enrichment

Since most of the metabolic genes with high regulatory load in macrophages are preferentially expressed in macrophages, and are usually the most abundantly expressed genes within their respective pathway, we asked in addition whether the positioning of the reactions they control within the macrophage metabolic network is also different from other reactions. Indeed, we could observe clear differences when focusing on the genes associated to transporters or entry points of the pathways predicted active in the macrophage model (Fig. 6). While 53.1 % of all gene-associated reactions in our macrophage metabolic model are transport or entry point reactions, this fraction increases significantly to 67.1 % when focusing on reactions associated to high regulatory load genes (KS-test, *p*-value = 9.0e-5). Furthermore, when looking only on transport reactions that constitute 17.4 % of all macrophage reactions, we observe an even more significant enrichment (KS-test, *p*-value = 1.8e-7) to 32.9 % of the reactions associated with high regulatory load. Finally, when excluding the transport reactions and focusing on the reactions corresponding to the remaining entry points of the different pathways (44.7 % of all macrophage reactions) we also see an enrichment for the high regulatory load genes (51.6 % of high regulatory load reactions), although with clearly higher p-value (KS-test, *p*-value = 0.0839). Importantly, similar results could not be obtained using a generic metabolic reconstruction such as Recon2 (data not shown), further highlighting the importance of using context-specific models and cell type-specific epigenomic data.

Taken together, genes associated to reactions at important control points of the macrophage metabolic network such as transporters or other pathway entry points are particularly enriched for high regulatory load, and exhibit abundant and cell type-specific expression patterns, possible enabling cell type-specific control of the downstream pathways.

### Entry to alternative bile acid synthesis pathway via CYP27A1 is under high regulatory load and depends on multiple transcription factors

**Fig. 7:**
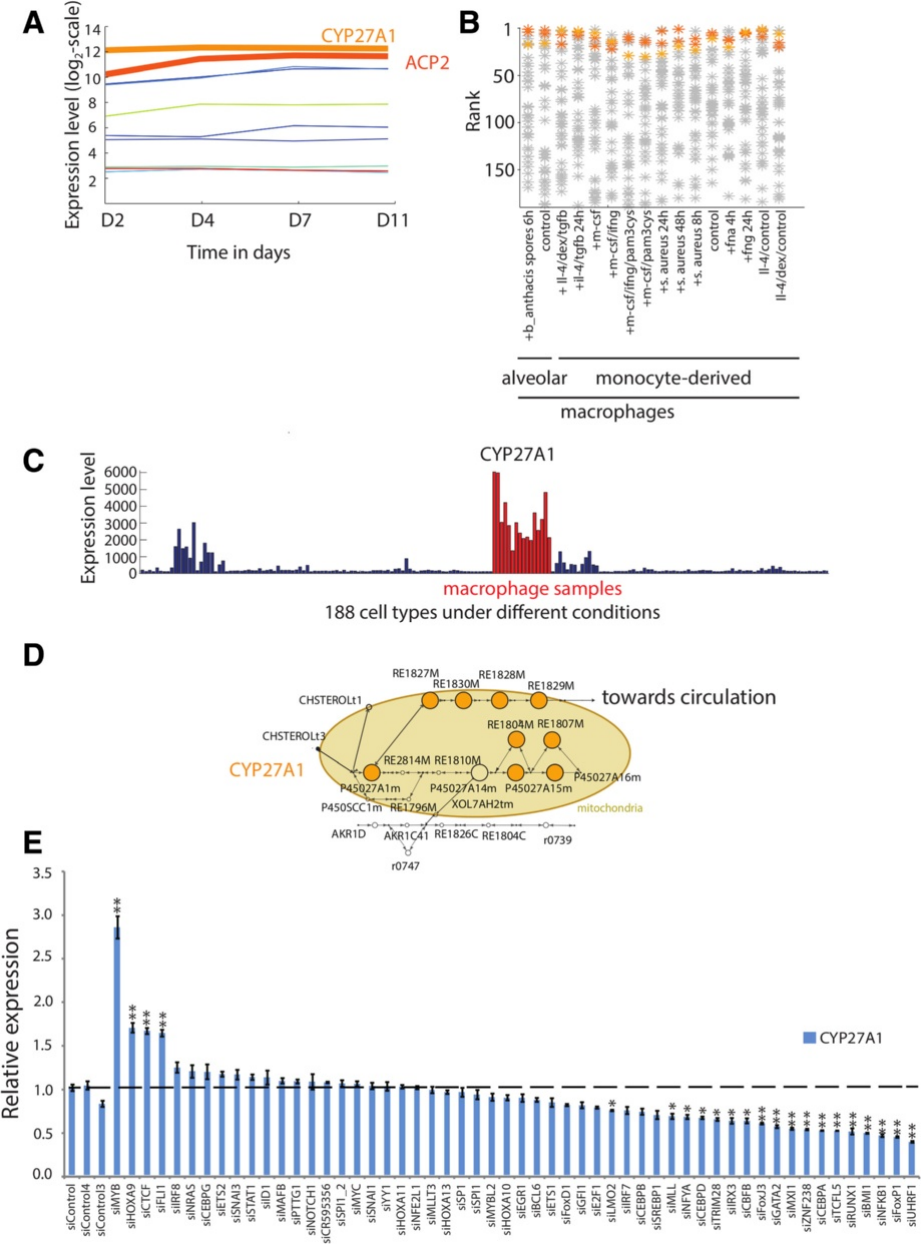
The alternative pathway of bile acid synthesis is controlled by high regulatory load on *CYP27A1* gene. **a** The mean normalized expression values of the genes implicated in the bile acid synthesis pathway based on the microarray data across the four differentiation time points are depicted. High regulatory load genes (*CYP27A1* and *ACP2*) are presented in different shades of orange and with a thicker line than other genes of the pathway. **b** For each gene of the bile acid synthesis pathway, the rank of the expression level in the macrophage samples among the 188 conditions and cell types of the Primary Cell Atlas are shown by an orange or gray star (*) for high regulatory load genes and genes that are not under high regulatory load, respectively. Genes in the top ranks are situated in the top of the figure. **c** The expression profile for *CYP27A1* across all 188 conditions and cell types from Primary Cell Atlas as arbitrary expression units. Macrophage samples are depicted in red. **d** The alternative pathway of bile acid synthesis was visualized in Cytoscape. To allow the alternative pathway to carry a flux, an exchange reaction was added, enabling the export of the last metabolite from the cell. Reactions predicted active in this modified macrophage model (day 11) are depicted as filled black circles or filled orange circles for reactions under control of high-regulatory load genes. The size of the nodes correlates with the number of associated enhancers. The reaction names correspond to reaction-identifiers of Recon 2. **e** The normalized expression levels of *CYP27A1* in microarray analysis of THP-1 monocytes in a series of knock-down experiments for 53 different transcription factors or regulators and three unspecific control siRNAs retrieved from the FANTOM consortium database. Expression values were normalized to the first control siRNA (siNC) and represent the mean expression values ± SD (n ≥ 3). Student’s *t*-test determined the significance of changes in response to siRNA transfection (*, *p* <0.05; **, *p* <0.01)

An interesting example among pathways with differential activity in macrophages is the bile acid synthesis pathway, which also serves as the major cholesterol catabolism pathway. Consequently, it also produces intermediates like oxysterols that serve as regulators of gene expression through their role as endogenous ligands for transcription factors like liver X receptors (LXRs). The bile acid synthesis pathway has two genes with high regulatory load in macrophages, CYP27A1 and ACP2, which are also the highest expressed genes of the pathway throughout the differentiation from monocytes to macrophages (Fig. 7a). Both genes are the most macrophage-specifically expressed genes of the pathway (Fig. 7b) and CYP27A1 shows the most abundant expression in different macrophage cells and selected dendritic cells (Fig. 7c). CYP27A1 is known to be involved in catalyzing the mitochondrial reactions of the classic, or neutral, bile acid synthesis pathway in the liver [42, 43]. In addition, CYP27A1 is also responsible for the first reaction of the alternative, or acidic, pathway to hydroxylate cholesterol directly in the mitochondria to 27-hydroxycholesterol in extrahepatic tissues, in particular in macrophages (Fig. 7d) [44]. Therefore CYP27A1 is a prime example of a high regulatory load gene potentially integrating multiple signals to control an entry point reaction of an alternative pathway.

Finally, to test which transcription factors could be responsible for the high regulatory load of *CYP27A1*, we analyzed microarray data from the FANTOM consortium for knock-down experiments of 53 transcriptional regulators in THP1 monocytes (Fig. 7e) [45]. Interestingly, almost half of the tested knock-downs affected *CYP27A1* expression directly or indirectly with 18 TFs showing significant downregulation after transfection and additional 4 regulators causing a significant upregulation (Fig. 7e). Among the TFs causing significant change in *CYP27A1* expression upon knock-down were many known myeloid regulators that were also predicted as key TFs based on our *de novo* motif analysis (Additional file 1: Figure S4), including CEBP-family members, Forkhead-family members, and FLI1. Moreover, CEBPB and SREBF1 knock-downs both led to decreased expression levels just above the significance cut-off with p-values of 0.055 and 0.054, respectively, altogether indicating that *CYP27A1* expression is controlled by multiple transcription factors in monocyte-derived macrophages.

## Discussion

Here we present a novel workflow, FASTCORMICS, for the fast, robust and accurate generation of metabolic models based on transcriptomics data generated by microarrays and use FASTCORMICS to generate multiple metabolic models across tens of primary cell types. This analysis reveals a cell type-specific usage of the alternative branches in metabolic networks and raises the question about the epigenetic regulation of metabolism in different cell types. To address this question we performed genome-wide mapping of active enhancers in primary human macrophages and integrated these data with metabolic models of monocyte-to-macrophage differentiation to expose the metabolic genes under high regulatory load in macrophages and general features of these genes within metabolic networks. Interestingly, the high regulatory load genes show the most abundant and cell type-selective expression profiles of the genes within any metabolic pathway and control in particular the different transport and entry point reactions of the pathways.

An interesting example of a metabolic enzyme controlling an entry point of an alternative pathway is CYP27A1, which is encoded by one of the 55 metabolic genes under high regulatory load in macrophages. The alternative bile acid synthesis, which is initiated by CYP27A1 in mitochondria, is also the major cholesterol catabolism pathway in macrophages. Therefore the regulation of *CYP27A1* can be used to control cholesterol homeostasis in macrophages, and other extra-hepatic cell types, on one hand through initiating cholesterol catabolism, and on the other hand due to production of intermediate oxysterols that indirectly influence cholesterol efflux and biosynthesis [46]. *CYP27A1* has therefore many implications to the development of atherosclerosis and cardiovascular disease. Moreover, a mutation of *CYP27A1* in humans causes a disease called cerebrotendinous xanthomatosis (CTX), which leads to accumulation of cholesterol in brain and tendons and is accompanied by neurological dysfunctions, including parkinsonism, as well as increased rate of atherosclerosis [47, 48].

The disease-association of *CYP27A1* is consistent with previous findings from us and others that genes under high regulatory load, or controlled by so called super-enhancers, are often associated with disease [40, 49]. Indeed, our current findings suggest that within any cell type the top 10^th^ percentile of highest regulated genes are significantly enriched for disease-association (which is also the reasoning behind the applied cut-off for high regulatory load in this study) [50]. This is possibly due to their central roles as network hubs within gene regulatory networks, forming integration points for multiple signals. While this combinatorial regulation can be robust, it might also increase the likelihood of being affected by alterations such as single nucleotide polymorphisms (SNPs) in the regulatory regions. This would be consistent with the experiments of Siersbaek et al. who showed that omission of one TFs activity, that of glucocorticoid receptor (GR), in early adipocyte differentiation had more potent effect on super-enhancer activity than on activity of more isolated GR binding sites [13].

Integrating gene regulatory networks with metabolic networks is an important and necessary step for truly global understanding of metabolism and its regulation. However, the role of high regulatory load genes in control of metabolism has not been previously specifically addressed. We find that high regulatory load genes, the central hubs of the gene regulatory networks, are significantly enriched for controlling transport reactions or other entry points of pathways, like in the case of *CYP27A1*, with almost 70 % of such reactions located at transporters/entry points (Fig. 6). They are the most abundantly expressed genes within the pathways and show most variation between cell types, suggesting they are used as the control points for cell type-specific metabolism. This is consistent with the findings in metabolic control analysis that for linear pathways with similar individual kinetics assigned to the different enzymes the flux control exerted at the upper part of the pathway and especially at the first step is much higher than in the lower part [51].

While most high regulatory load genes do control entry point reactions, there remains a large proportion of them that do not. An interesting question is what other network positions are controlled by high regulatory load and to which end. Among the non-entry point reactions associated to high regulatory load genes in macrophages many are situated immediately downstream of branch points where a metabolite can follow two different fates within the pathway. For example, kynurininase (KYNU) is a high regulatory load gene catalyzing branch point reactions in tryptophan metabolism pathway to decide the faith of tryptophan metabolite kynurenine into downstream metabolites with inflammatory and neuroactive functions [52]. Similarly, UDP-glucose ceramide glucosyltransferase (UGCG) is a macrophage high regulatory load gene controlling the commitment of sphingolipids to glycosphingolipid branch [53]. Interestingly, the enzyme is also required for capture of HIV-1 viral particles into dendritic cells and useful for the virus upon infection [54]. In addition to branch point reactions, many high regulatory genes also control reactions along the metabolic pathways. Regulation at such positions might be important for example to control accumulation of harmful or beneficial metabolic intermediates. However, it should also be pointed out that the consistency of the current human GENREs like Recon 2 is only approximately 75 % and many branch or entry points might still remain unannotated.

In general the context-specific reconstruction of metabolic network models with FASTCORMICS as presented here might be severely influenced by the quality of the used GENRE, especially when applying automated annotation pipelines. As the overall run-time of FASTCORMICS is very low, it allows performing cross-validation studies as described earlier and thereby detecting high-confidence reactions with multiple evidence for their presence in the context-specific model of interest. In general, with run-times in the order of seconds FASTCORMICS clearly out-performs competing algorithms and might serve as an important corner stone of many future applications.

We’ve used FASTCORMICS to generate metabolic models of hundreds of human cell types, including a time-course of monocyte-to-macrophage differentiation. As discussed above, the cholesterol metabolism was predicted to be increased between day 2 and day 11 of the differentiation (Fig. 3), consistent with the ability of healthy resident macrophages to uptake and release lipids, as part of their generic cleaning role or in a targeted way through low density lipoproteins (LDLs) [55, 56]. This may also be correlated to the observed increase in the active reactions in phospholipid (more precisely glycerophospholipids in Fig. 3) metabolism or overall increase in expression of genes associated to reactions in triacylglycerol synthesis, the two other main lipid families that constitute LDLs. Also, the differentiation process between day 2 and 11 predicts an increase in the metabolism of the essential amino acid tryptophan, in particular with respect to its kynurenin metabolite [57]. In addition, also the metabolism of other relevant metabolites like the eicosanoids, another important signaling family [58], or glutamate [59], were increased, as well as pathways with fewer specific implications for macrophage biology like inositol phosphate, pyruvate and propanoate metabolisms. FASTCORMICS is therefore able to contextualize a qualitative and quantitative difference between monocytes and macrophages.

More detailed analysis of pathophysiologic states of monocyte-to-macrophage differentiation in inflammatory conditions could be another informative application of the predictive efficacy of FASTCORMICS. Indeed, inflammation of the vascular wall is for example disturbing the uptake and release equilibrium of lipids by macrophages, making them become lipid-loaded foam cells by mechanisms involving oxidized LDL, and thus participate to the development of atherosclerosis [56]. Also, inflamed microglia (the resident brain macrophages) have been shown to produce enhanced quantities of quinolinic acid, a metabolite of the tryptophan-derived kynurenin, which can become toxic to the brain and could participate to the development of various neurodegenerative processes among which Alzheimer’s and Parkinson’s diseases [52, 60].

FASTCORMICS allows in a modular fashion to use medium information and/or a biomass function for improved contextualization. This would allow generating more accurate context specific network models. However, it might be challenging to obtain specific medium and biomass information for reconstructing a cell’s metabolism residing within a multi-cellular context. In the presented work a general biomass function was used. Future progress in the respective analytical methods will therefore help to further improve the contextualization via FASTCORMICS by providing more accurate specific medium and biomass information.

FASTCORMICS is based on the discretization of the expression data with Barcode, which to our knowledge currently is the most robust and reliable discretization method. The pre-processing step with Barcode allows circumventing the need of setting an arbitrary expression threshold that segregates between expressed and non-expressed genes as e.g. in [21, 23, 24]. As such a threshold is arbitrary and critical for the output metabolic models as in response to this threshold complete branches, alternative pathways, or subsystems might be included or excluded, thereby heavily changing the functionalities of the model. Further, Barcode shows a better correlation between predicted expression and protein expression than competing discretization methods for the segregation of gene expression and allows reducing batch and lab-effects that affect measurements [30].

An interesting future research question is if better context-specific reconstruction could be obtained by applying continuous weights instead of discrete core assignments or by a combination of the two approaches. While in general continuous weights might be able to better capture the continuous distribution of expression values, this would require the setting of arbitrary parameters to convert expression values into optimization weights, thus rendering this approach biased to arbitrary settings as also stated by Machado et al. [61]. Thus the overall performance of such approach needs to be investigated in more detail in future work. FASTCORE can form a valuable building block here as well.

Such continuous approach might also be suitable to treat genes with reactions associated in multiple pathways (like the discussed *CYP27A1* example) more efficiently, where a stringent including of core reactions without integration of the expression context of the remaining reactions in the pathway might not be the best approach.

Furthermore FASTCORMICS can easily be adapted for the integration of other omics types, like data from next generation sequencing methods such as RNA-seq, while special attention has to be paid to the data type specific discretization step.

## Conclusion

FASTCORMICS allows obtaining high-quality, robust models in a high-throughput manner. This allows the use of metabolic modelling as routine process for the analysis of expression data. Further integration with gene regulatory network data opens possibilities for better understanding of the upstream events and identification of novel drug targets such as the genes under high regulatory load which we here find to control entry points of pathways in the macrophage metabolic network.

## Methods

### Building of context-specific models with the FASTCORMICS workflow

The general workflow of FASTCORMICS (Additional file 1: Figure S1) contains a discretization step with Barcode to obtain for each gene a z-score which indicates the number of standard deviations of the gene of the considered array above the mean expression value of the same probe set in an unexpressed context measured across thousands of arrays. Genes with a z-score equal or below zero, corresponding to the mean of the distribution of the non-expressed genes, are considered as inactive and are associated with a discretization score of −1. Genes with z-score above 5, corresponding to the threshold value benchmarked by Zilliox et al. [30], are considered as expressed and get a discretization score equal to 1. Genes with z-score larger than 0 but smaller than 5 form the undetermined gene set and get a discretization score of zero. The discretization score 1 is then mapped to the consistent generic model via the model’s Gene-Protein-Reactions Rules (GPR) to obtain a list of active reactions (core reactions). For reactions that are under the control of one gene only, the discretized gene score is directly mapped to the reaction. If more genes are associated to a reaction, the relationship between the genes and the reaction is given by Boolean Rules. A Boolean AND means that all the genes have to be expressed to activate the reaction, which is typically the case when a reaction is controlled by a complex of proteins. Therefore the minimum of the discretization score is mapped to the reaction. A Boolean OR signifies that only one gene has to be expressed. The maximal discretization score value is then mapped to the reaction. Boolean ANDs and ORs can be combined inside the same rule, e.g. ((A AND B) OR C), in this example the minimal value D is computed of A and B, and then the maximum between D and C is matched to the reaction. Reactions associated to a discretization score of −1, are considered as inactive and removed from the model by setting their bounds to zero. Reactions with a discretization score of 1, form the set of core reactions that are fed into a modified version of FASTCORE (mFC) that allows leaving a set of reactions non-penalized besides defining core and non-core reactions. The inclusion of non-penalized reactions is, unlike core reactions, not forced, but only preferred over the inclusion of penalized non-core reactions. Barcode-supported transporters are put to the set of non-penalized reactions. Transport reactions are generally under the control of promiscuous genes (in the consistent version of Recon 2 e.g. the gene SLC7A6 controls 294 reactions) and therefore transporters are not included into the core set as otherwise whole subsystems would be included in the output model due to one gene. Nevertheless, the inclusion of Barcode-supported genes should be preferred over non-core reactions (which are not supported) and therefore Barcode-supported transporters are not penalized. For more details on FASTCORE see the original paper [28]. A MATLAB implementation of the FASTCORE and FASTCORMICS algorithms will be available for download from bio.uni.lu/systems_biology/software.

Three optional steps can be included in the workflow. The first one allows further constraining the model with respect to the medium composition, if this information is available. Uptake reactions for metabolites not being present in the medium are shut down and FASTCC [28] is run to remove reactions that cannot carry a flux due to these additional constraints. The second optional step allows adding a biomass function or of production reactions of specific metabolites to the model. FASTCORMICS forces the biomass function or/and the corresponding exchange reactions to carry a flux while penalizing the inclusion of non-core reactions (Additional file 1: Figure S1). Core reactions, including core transporters, are not penalized in order to find, within the different alternatives sets of reactions that allow the production of biomass or required metabolites, the one that contain the highest number of core reactions. The output reactions of the modified FASTCORE are then added to the core set and the modified FASTCORE is run a second time to now force all the core reactions to carry a flux while penalizing the non-core reactions. Transport reactions are removed from the core set, but are not penalized during the reconstruction to favor Barcode-supported transporters over non-core reactions that are not supported. If no biomass function is added, FASTCORMICS is only run once. Finally a cross-validation step can be performed to assign a confidence score to the reactions included in the model. For the latter, the building process is repeated multiple times, leaving at each run one core reaction out. Reactions (core and non-core reactions) present in all the runs are supported by at least 2 core reactions and therefore are assigned a high confidence score, whereas core reactions that were not recovered during their left-out run are supported by the expression value of their own gene(s) only. The remaining non-core reactions have a low confidence score as they themselves are not supported by Barcode and their inclusion in the model depends on a single core reaction only. The same process can also be repeated with the non-expressed reactions set in order to estimate if a sub-branch of a pathway was removed from the model due to the presence of a single unexpressed reaction or to multiple inactive reactions that interrupts the flux.

### Reconstruction of generic cancer models

The NCI dataset composed of 174 Hgu133plus2 arrays corresponding to 59 cancer cell lines was downloaded from the Cell miner web page [31] and read in R version 2.15.1 using the affy package (1.36.1). The arrays were normalized with the frozen Robust Multi-array Average package (fRMA version 1.14.0) [62] using the core target and the median polish option. The normalized values were then processed with Barcode using the hgu133plus2frmavrecs vector (version 1.1.12) into a list of probe sets IDs with the respective z-score (Additional file 1: Figure S1). The list of probe sets was then converted in Entrez IDs via the hgu133plus2.db package (Carlson M. R package version 3.0.0). The z-scores are converted into discretization scores (1, 0, −1) using the above mentioned expression threshold of 5 and non-expression threshold of 0. The ubiquity of expression (sum of the discretization score for a gene over all arrays) was computed for each gene and a list of genes Entrez IDs with their respective score was then loaded in Matlab (version 2013a) and mapped via the Gene Protein Reactions Rules (GPR) to the consistent version of Recon1 (consistRecon1, 2469 reactions) and Recon2 (consistRecon2, 5317 reactions, the lower bound of the AATAI reaction was set to zero to be consistent with the reversibility information of the model) obtained with FASTCC. To be consistent with the experimental setup of Folger et al. [21] reactions tagged as active in ≥90 % of the 174 arrays were included in the core set with the exception of Barcode-supported transport reactions. Reactions with ubiquity of expression score equal below zero in ≥90 % were removed from the model as explained previously. To be comparable to the results of Folger et al. and Luo et al. [21, 33] the growth of the cancer cells was simulated on RPMI medium, the uptake reactions of the consistent versions of Recon 1 and Recon 2 were constrained with respect to the medium composition (Additional file 2: Table S2, medium composition sheet). Uptake reactions for the metabolites present in the medium were automatically added within FASTCORMICS if required by the biomass function taken from Wang et al. or for the inclusion of a barcode-supported pathway. Beside a biomass function, a sink reaction was added to Recon 1 to balance the glycogenin self-glucosylation reaction [21, 33]. The exchange reaction of triacyglycerides in Recon 2 was left unconstrained. FASTCC was run to remove reactions that are not able to carry a flux due to these additional medium constraints (Additional file 1: Figure S1).

The modified FASTCORE was then run on the medium-constrained models forcing the biomass function to carry a flux while penalizing the inclusion of non-core reactions. The reactions required to allow a biomass production were then added to the core set and the modified FASTCORE was run again now forcing the inclusion of all core reaction while penalizing the non-core reactions with the exception of core transporters.

The pre-processing step with Barcode for large data sets was performed due to memory issues on a Linux compute server with 3.0 GHz Intel Xeon CPU and 16 GB RAM and took 3 min. The model reconstructions were performed on a standard 3.40 GHz Intel Core i5 computer with 4 GB RAM in 38 and 288 s for cancer 1 and cancer 2 respectively, so that the overall computational time of the FASTCORMICS workflow is below 5 min.

### Validation of the cancer models by comparison to an shRNA screen on cancer cell lines

A *in silico* knock-out experiment was performed on the obtained cancer models as previously described by Folger et al. applying Flux Balance Analysis (FBA) [26, 63]. In Folger et al. a gene is considered essential if its knock-downs results in a decrease of the growth rate of more than 1 %. To allow, a comparison with Folger et al. the 1 % criteria was kept. The lists of essential genes were compared to the ranked list of 8000 genes established by Luo et al. based on an shRNA knockdown screen on cancer cell lines. The rank of the essential metabolic genes were compared to the rank of the remaining metabolic genes (set of genes associated to Recon2 minus the essential genes) with a Kolmogorov-Smirnov test (KS-test). In addition 1,000,000 random sets of genes of the same size were created and the respective KS-test was computed for evaluating the likelihood to obtain the same or better KS-score by chance (Additional file 1: Table S1).

To further validate the predicted essential genes, a list of neoplasia-related genes was retrieved from DisGeNET, a database for gene-disease associations [21, 34]. A hypergeometric test was performed to evaluate the enrichment of neoplasia-related genes in the predicted essential genes (Additional file 1: Table S3).

### Reconstruction of 156 context-specific models of selected primary cells

The Primary Cells Atlas (GSE49910) gathering 745 arrays of the HG-U133_Plus_2 platform taken from 100 separate studies, corresponding to >180 different experimental conditions in tens of primary cell types, was downloaded from the Gene Expression Omnibus repository [35]. 156 arrays corresponding to 63 cell types were selected favoring control samples in order to derive undisturbed cell-specific metabolic pathways in resting cells (see Additional file 3: Table S4 for the list of selected arrays). The arrays were normalized with fRMA using the median polish and core target option and then discretized with the Barcode package (as in Reconstruction of generic cancer models). The probe set IDs were converted to Entrez IDs with the (hgu133plus2.db) package as above, which were mapped to the consistent version of Recon2. 156 models (one model per array) were built using the previously described FASTCORMICS workflow. The high efficiency of FASTCORMICS allowed to perform this task within 4.5 h (5 min for the pre-processing with Barcode on 3.0 GHz Intel Xeon CPU and 4.5 h for the model reconstructions on a standard 3.40 GHz Intel Core i5 computer with 4 GB RAM).

The primary context-specific models were represented as a matrix of 5317 rows corresponding to the reactions of the consistent Recon2 version and 156 columns for the number of models. The presence of the reactions in the different models was indicated by ones and the absence by zeros. The level of similarity between the different models pairs was determined via the Jaccard index. The resulting Jaccard index matrix of size 156 times 156 was then clustered with the MATLAB clustergram function (Fig. 1a).

### Isolation of primary human monocytes from blood

Primary human monocytes were extracted from the blood samples of anonymous healthy male donors, donated by the blood transfer centre of the Luxembourgish Red Cross and were used for diverse experiments in agreement with the convention between the Luxembourgish Red Cross and the University of Luxembourg from 16.05.2011 and following the principles of Helsinki Declaration.

The blood was diluted 1:1 with phosphate buffered saline (PBS) (Invitrogen, Life Technologies). Afterwards the peripheral blood mononuclear cells (PBMC), were isolated by Ficoll density gradient separation. Therefore the blood-PBS suspension was transferred to leucosep tubes (Greiner Bio One,) containing 15 ml of ficoll (VWR). After a 10 min centrifugation (1000x g, room temperature, without break), the mixture separated into an upper phase of plasma, followed by the white peripheral blood mononuclear cell (PBMC) layer, the separation gel ficoll and erythrocytes in the bottom of a 50 ml tube. The PBMC layer was collected and washed twice with ice-cold MACS buffer {(PBS, pH 7.2; 0.5 % bovine serum albumin (BSA) (Sigma-Aldrich, Seelze, Germany) and 2 mM ethylenediaminetetraacetic acid (EDTA) (Sigma-Aldrich, Seelze, Germany)} at 4 °C for 10 min at 300 g. From this step on cells were kept on ice. Following the separation of the PBMCs the CD14^+^ cells (monocytes) were isolated from the total PBMC fraction by using the MACS® technology from Miltenyi Biotec. In this method, anti-CD14^+^-antibodies are conjugated with superparamagnetic particles {CD14 MicroBeads (Miltenyi Biotec)} and bind to the CD14 antigen on the cell surface of CD14+ cells. By using a magnet {MACS separator (Miltenyi Biotec)} and LS Columns (Miltenyi Biotec) the CD14+ cells can be separated from the rest of the PBMCs. Before the CD14^+^ cells were separated, the PBMCs of one blood preservation were mixed with 200 μl of CD14 MicroBeads and incubated for 30 min at 4 °C on a rotating wheel. Afterwards the cells were washed with MACS buffer and centrifuged at 300 g for 10 min at 4 °C. The cells were again suspended in MACS buffer and loaded on a pre-washed LS-column which was put on a MACS separator, and contained a pre-separation filter (Miltenyi Biotec) on top, in order to avoid a blocking of the column. Subsequently the column with the CD14^+^ cells was washed and the CD14^+^ cells were eluted from the column with MACS buffer, after taking away the MACS separator.

### Differentiation of primary human monocytes into macrophages

After the successful isolation of the CD14^+^ monocytes, the cells were counted and seeded in a density of 2 × 10^6^ cells/ml, either in a 10 cm^2^ plates (of about 20 × 10^6^ cells) (Thermo scientific) in order to perform ChIP experiments or in 6-well plates (of about 4 × 10^6^ cells/well) (Thermo scientific) to extract RNA. For culturing and differentiation of monocytes to macrophages RPMI 1640 medium (VWR) was supplemented with 10 % human serum {off the clot, type AB (A&E Scientific, PAA, Pasching, Austria, lot number: C02108-1021)}, 0.1 mg/ml streptomycin (Invitrogen, Life Technologies), 100 U/ml penicillin (Invitrogen, Life Technologies) and 0.1 mM L-glutamine (Invitrogen, Life Technologies). The cells were kept at 37 °C under a 5 % CO2 atm. The medium was changed, during the differentiation process of monocytes to macrophages, 4 and 7 days after seeding. For the RNA extraction and the subsequent array analysis, the cells were extracted 2 days, 4 days, 7 days and 11 days after seeding (see Fig. 2). In order to perform ChIP experiments the chromatin of day 11 cells was cross-linked (see Fig. 2).

### Morphology of primary human monocytes and macrophages by microscopy

The morphology of the monocytes and macrophages was visualized by using the microscope Axiovert 40C (Zeiss) with a magnification between 10x and 20x, the camera AxioCAM MRC (Zeiss) and the software Zen blue (Zeiss). Unstained cells were used to generate pictures of the monocytes, macrophages and intermediate states (see Fig. 2).

### Total RNA extraction

The RNA was extracted by using TRI Reagent (Sigma-Aldrich). The cells in the 6-well plate were lysed with 500 μl of TRI Reagent per well. Following complete lysis, 100 μl of chloroform (Sigma-Aldrich) were added to the lysate, vortexed for 20 s and incubated at room temperature for 3 min. These steps were followed by 15 min centrifugation at 4 °C with full speed, during which the mixture separated into different phases, with the upper phase containing the RNA. This RNA containing phase was mixed with equal volume of ice-cold isopropanol (Sigma-Aldrich) in order to precipitate the RNA overnight at −20 °C to recover also all small RNAs. The pelleting of the RNA was done at full speed for 20 min at 4 °C. Then the RNA was washed with 70 % ice-cold ethanol (VWR) and centrifuged for 5 min at full speed and 4 °C. Finally, the RNA pellet was dried and solved in RNase-free water. The concentration and the purity of the RNA were measured with the NanoDrop 2000c (Thermo scientific). The quality of the RNA was measured with the 2100 Bioanalyzer from Agilent Technologies and all the RNA samples had a RIN number greater or equal to 8.

### Data analysis of mRNA microarrays

One hundred ng of total RNA was used to process Affymetrix Human Gene 1.0st microarrays. The Ambion® WT Expression Kit was used to reverse transcribe the RNA into cDNA and to purify it according to manufacturer’s instructions (The Ambion® WT Expression Kit Protocol For Affymetrix® GeneChip® Whole Transcript (WT) Expression Arrays Part Number 4425209 Rev.B 05/2009). Then the cDNA was fragmented, labeled and hybridized on the arrays according to The GeneChip® Whole Transcript (WT) Sense target Labeling Assay Manual Version 4 from Affymetrix (P/N 701880 Rev.4). The arrays were washed and scanned after 16 h of hybridization.

Microarray data were analyzed using Partek® Genomics Suite™, R Software (http://www.R-project.org/). First, 15 CEL files containing raw probe intensities were imported into Partek and data were preprocessed using the robust multi-array average (RMA) algorithm [64]. Preprocessing aims at estimating transcript cluster (gene) expression values from probe signal intensities. Boxplot and relative log expression calculated on resulting gene expression values were then used to assess the quality of data; no outlier was found. Principal component analysis (PCA) was then performed for data reduction and factor analysis. PCA was able to separate data according to the time. According to this observation, the Linear Models for Microarray (Limma) [65] package was used to identify genes for which expression changed throughout the time. Gene expression values were imported into R, Limma was applied and all times were compared to the gene expression values generated from D2 cells. Resulting p-value was adjusted for multiple testing errors using false discovery rate (FDR) [66]. The microarray expression data can be found at ArrayExpress (http://www.ebi.ac.uk/arrayexpress/) with accession number E-MTAB-3089.

### Reconstruction of the monocyte-macrophage models

The 15 microarrays of the Hugene.1.0.st.v1 platform were read into R version 2.15.2, with the oligo package (1.22.0) and normalized with the fRMA package (1.14.0) and the hugene.1.0.st.v1frmavecs (1.0.0) vector and then discretized with Barcode. The probe sets were converted in Entrez ID via the hugene10sttranscriptcluster.db package (MacDonald JW. R package version 8.2.0). The discretized values were then mapped to the consistent version of Recon 2 (version 3, the lower bound of the AATAI reaction was set to zero to be consistent with the reversibility information of the model). In order to minimize the effect of patient-specific variation on the models, reactions tagged as active in the cells of 3 out of 4 donors for each time point, respectively 2 out of 3 for time point D4 were included in the core set, with the exception of the core transporters that were removed from the core set, but not penalized during the building process. Similarly, reactions tagged as inactive in 3 out of 4 or 2 out 3 donors were removed from the models as explained previously. Cross-validation was used to determine the confidence levels of the included and excluded reactions. Reactions with a high level of confidence are supported by at least two core reactions. Reactions with moderate confidence level are reactions only supported by barcode. Reactions with a weak confidence level are not supported by expression, but needed to generate a consistent network model. Excluded reactions with a high confidence score were never included in any simulations suggesting the presence of other excluded reactions in the branch. Whereas, excluded reactions with a low confidence level were excluded only due to their low expression level.

### Chromatin immunoprecipitation (ChIP)

The primary human macrophages (15.5-21 × 10^6^ cells/10 cm^2^ dish) were fixed for 8 min with 1 % formaldehyde in PBS (Sigma-Aldrich) and were washed before with PBS. Then the formaldehyde was quenched for 5 min with a final concentration of 125 mM of glycine (Sigma-Aldrich). The fixed cells were washed twice with PBS, the PBS of the second washing step contained protease inhibitor (PI, Roche Applied Sciences). This step was followed by scraping the primary human macrophages in the PBS-PI solution and spinning them down at 4 °C for 5 min at 1300 rpm. The pellet was resuspended in 1500 μl of ice-cold lysis buffer (5 mM 1,4-piperazinediethanesulfonic acid (PIPES) pH 8.0 (Sigma-Aldrich); 85 mM potassium chloride (KCl) (Sigma-Aldrich); 0.5 % NP-40 (VWR)) containing PI and incubated for 30 min on ice. Afterwards, the cell lysate was centrifuged at 5000 rpm for 10 min at 4 °C. The pellet was resuspended in 750 μl of ice-cold shearing buffer {50 mM Tris Base pH 8.1 (Sigma-Aldrich); 10 mM EDTA, disodium salt (Sigma-Aldrich); 0.1 % sodium dodecyl sulfate (SDS) (Sigma-Aldrich); 0.5 % sodium deoxycholate (Sigma-Aldrich,)} into which fresh PI was added. After 30 min incubation on ice, the chromatin was sheared with a sonicator (BioruptorTM Next Gene, Diagenode) during 30 cycles at high intensity (30 s off and 30 s on). The sheared chromatin samples were then centrifuged at 15.000 rpm for 10 min at 4 °C in order to pellet the remaining cell debris. The supernatant, which contains the chromatin, was transferred to a new tube.

Twenty-five μl of the sheared chromatin was purified to check the size of the sheared DNA on an agarose gel. The concentration of the DNA was determined by the Qubit dsDNA HS Assay Kit (Invitrogen) and the Qubit 2.0 Fluorometer (Invitrogen) according to the manufacturer’s instructions.

For each immunoprecipitation 5 μg of sheared chromatin and 0.5 μg as input were used. In order to pre-clean the chromatin, the sheared chromatin was diluted with modified RIPA Buffer {(140 mM NaCl; 10 mM Tris pH 7.5; 1 mM EDTA; 0.5 mM ethylene glycol-bis(2-aminoethylether)-N,N,N′,N′-tetraacetic acid (EGTA) (Sigma-Aldrich); 1 % Triton X-100 (Sigma-Aldrich); 0.01 % SDS; 0.1 % sodium deoxycholate (Sigma-Aldrich)} containing PI, up to 1200 μl, and incubated for 30 min with 25 μl of protein A magnetic (PAM) beads (Millipore) at 4 °C on a rotating wheel. Afterwards, the PAM beads were captured with a magnet and the supernatant containing the pre-cleared chromatin was transferred to a new tube. This pre-cleared chromatin was then incubated overnight with 5 μg of an antibody against the active enhancer mark H3K27ac (Abcam, product No.: ab4729). On the next day the antibodies were captured with 25 μl of PAM beads during 2 h on a rotating wheel at 4 °C. This step was followed by pelleting the magnetic beads on the tube side by using a magnetic stand. The supernatant was discarded and the PMA beads, linked with the antibodies and, due to this, to chromatin, were washed twice with 800 μl of wash buffer 1 {(20 mM Tris pH 8.1; 50 mM NaCl; 2 mM EDTA; 1 % TX-100 (Sigma-Aldrich); 0.1 % SDS)}, once with 800 μl Wash Buffer 2 {(10 mM Tris, pH 8.1; 150 mM NaCl; 1 mM EDTA; 1 % NP40; 1 % sodium deoxycholate (Sigma-Aldrich); 250 mM lithium chloride (LiCl) (Sigma-Aldrich)} and twice with 800 μl TE buffer (10 mM Tris pH 8.1; 1 mM EDTA pH 8). All the washing steps were performed for 2 min on a rotating wheel at room temperature, followed by pelleting the beads on a magnetic stand. In order to detach the chromatin from the PMA beads and to get rid of the proteins, the washed beads as well as the input were incubated with 100 μl elution buffer (0.1 M sodium bicarbonate (NaHCO3) (Sigma-Aldrich); 1 % SDS) and 10 μg RNase at 65 °C overnight on a shaking platform. 5 μg of proteinase K were added after the overnight step for 90 min at 42 °C. Afterwards, the DNA was purified with a QIAquick PCR clean-up kit. Again, the DNA concentration was measured by using the Qubit dsDNA HS Assay Kit and the Qubit 2.0 Fluorometer according to the manufacturer’s instructions.

### ChIP-Seq

ChIP-Seq was performed with chromatin from three different donors. For each donor one ChIP sample using an antibody against H3K27ac and one input sample were sequenced. The sequencing of the ChIP samples was done at the Genomics Core Facility in EMBL Heidelberg. For sequencing, single-end-reads were used and the samples were processed in an Illumina CBot and sequenced in an Illumina HiSeq 2000 machine. The sequencing data can be found at Gene Expression Omnibus GEO (http://www.ncbi.nlm.nih.gov/geo/) with accession number GSE68798.

### Quality control and identification of enriched genomic regions

After sequencing the quality of the raw reads was controlled by applying the software FastQC v.0.10.1 (http://www.bioinformatics.babraham.ac.uk/projects/fastqc/). The reads that had a low quality base pair calling or the ones, which were detected as read artefacts were removed from the dataset (minimum quality score of phred 10 across the read length was required). Furthermore, these reads were read stacks collapsed using the FASTX software v.0.0.13 (http://hannonlab.cshl.edu/fastx_toolkit/index.html). The reads, which were not rejected by the quality control, were aligned to the human genome version 19 (hg19). This was done by applying the software Bowtie v0.1.25 [67] (one mismatch allowed, maximum three locations in the genome from which the highest quality match was reported).

The software QuEST v.2.4 [68] was used in order to identify enriched regions. The 44-mers were aligned to the hg19 by using the mappability parameter 0.88. The ChIP enrichment was set to 15 and the ChIP to background enrichment to 3. BigWig files were generated, which were used to visualize the data with the software Integrated Genome Viewer (IGV) v.2.3 (http://www.broadinstitute.org/software/igv/home) [69] (Fig. 4).

### Generation of enhancer-to-gene associations

The identified enriched regions were extended to both sides to create sequences of 450 bp in length that would include the sequences immediately flanking the modified histone and thereby capture the potential TF binding sites. Afterwards the lists of enriched regions were overlapped, generating a list of 16290 common enriched regions. For this the software Galaxy (http://galaxyproject.org) (07.07.2015, version 15.05) was used. Afterwards the common enriched regions were analyzed for their association to all genes by using the software GREAT v.2.0.2 [70] with the setting “single nearest gene within 500 kb”. For the metabolic genes these associations were further manually curated to make sure that the identified loci do not contain alternative highly expressed genes or previously unannotated transcripts such as non-coding genes.

For *de novo* motif analysis only enhancers associated to genes upregulated ≥2-fold in macrophages (D11 cells compared to D2 cells) with a FDR < 0.05 were considered. These were derived independently of the GREAT analysis by taking the TSSs of the up-regulated genes and extending +/−200 kb and overlapped with the common enriched enhancer regions by using Galaxy (http://galaxyproject.org).

### *De novo* motif identification

In order to identify the common sequences of the putative enhancers associated to genes upregulated in macrophages, *de novo* motif identification was performed by using the software MEME-ChIP (http://meme-suite.org/tools/meme-chip) (08.07.2015) version 4.10.01 [71]. Database Jolma 2013 [72] for known TF binding motifs was used to identify the TFs that might have bound to the putative enhancers. For the analysis of the data default settings were applied. For the MEME options the expected motif site distribution was set to zero or one occurrence per sequence. The count of motifs was set to 10. The minimum width of the motifs was set to 6 bp and the maximum width to 25 bp. For the CentriMo analysis, the software was asked to find uncentered regions and include sequence IDs.

These analyses generated a list of enriched motifs, which were linked to TFs that potentially bind these motifs. The enriched motifs were identified by analysing the meme-chip.html data generated by the MEME-ChIP software. Therefore, the e-value generated for the motifs found by the different programs were considered. The motifs with a known TF binding site are listed in the Additional file 1: Figure S4. The motifs with an *e*-value < 0.05 were considered. In addition, if a TF was associated to a motif that occurred several times, the motif with a lower e-value was considered.

### Test for specific location of high-regulatory load genes at entry points of pathways

The enrichment of transport reactions under high-regulatory load among gene-regulated reactions was computed via a hypergeometric test. For this test, the population size N, the number of successes states in the population K, the number of draws n and the number of successes k are respectively equal to the number of reactions in the macrophage model under gene control, the number of transporter under gene regulation, the number of genes under high-regulatory load and the number of transporter under high-regulatory load. In Recon 2, most transporters reactions were assigned to so-called transport subsystems (i.e. transport nuclear) but nevertheless some transporters are part of other pathways. For this study, we defined transporters as reactions that carry metabolites between compartments and therefore both types mentioned above were included.

To investigate if others reactions, beside transporters, were under high regulatory load, an enrichment test for reactions under the control of high-regulatory load genes situated at entry points of pathways was performed. An entry point is defined as the first reaction after a pathway change as annotated in the input models. Thereby the flux direction is taken into account. To identify entry points of pathways, for each reactions of the macrophage model under gene control flux variability analysis was performed to determine in which direction the reaction can carry a flux. It is important to note that the reversibility of the reactions given by the bounds only partially addresses this question, as the reversibility of adjacent reactions constraint the overall flux direction as well. Consumed metabolites in each reaction were identified in order to determine if one or more reactions producing these metabolites were part of a different pathway. Inorganic metabolites, CO2, known cofactor combinations (see Additional file 6: Table S8) and metabolite couples that do not change during the chemical reaction and therefore have the same chemical formula, were not considered. Reactions only composed of metabolites defined previously as cofactor are not taken into consideration as this would lead to a high numbers of false positives. Further Acetyl-coA is considered as a co-factor if it acts as a coA donor in the reaction. For each of the remaining metabolites, the producing reactions and the pathways to which they belong were determined. If the producing reaction is not a transporter and belongs to a different pathway than the considered reaction, the latter is an entry point of the pathway. In case that the producing reaction is a transporter, the transported metabolites and initial compartment were determined. If the pathway is the same as the considered reaction, the latter is an entry point after a compartment change, otherwise it is an entry point after a pathway change.

To avoid a bias of the enrichment test due to the transporters reactions, the latter were not considered for the test and therefore the population size N was defined as the number gene regulated reactions in macrophage being not part of the transporter set. The success in population K was defined as the set of reactions being entry points, the number of draw n are the reactions being entry points while excluding transporters and the successes k are the entry points under high regulatory load. The test was repeated without excluding transporters and successes were then defined as transporters or entry point reactions under high regulatory load. Finally, pathways were visualized in Cytoscape via the outputNetworkCytoscape function of the Cobra toolbox in MATLAB.

## Additional files

Additional file 1:
**FASTCORMICS allows fast context-specific metabolic model reconstruction using microarray data.** This files furthermore contains: **Table S1**. Essentiality testing of different cancer models, **Table S3**. Hypergeometric test quantifying the enrichment of neoplasia related genes retrieved from DisGeNet, **Table S6**. Summary of the monocyte-macrophage models, **Table S7**. Confidence level of the included and excluded reactions of the monocytes macrophage models, as well as **Figure S1**. FASTCORMICS workflow, **Figure S2**. Correlation plot of predicted lactate secretion rates by context-specific cancer cell models and measured lactate secretion rates, **Figure S3**. Scatterplot of the fraction of active reactions in monocyte derived macrophages versus the fraction of active reactions in monocytes. (PDF 2196 kb) Additional file 2: Table S2. Medium composition and biomass formulation. The biomass equations are given with the metabolites abbreviations IDs as found in the models (sheet 2). In the sheet three, the 1st column contains the medium composition used to constrain Recon 1 using the metabolites abbreviation as used in the model. In the second column are displayed the medium composition for Recon 2. (XLSX 12 kb) Additional file 3: Table S4. List of selected 156 arrays ordered in function the Jaccard similarity index used for Fig. 1 and of the remaining 745 arrays that compose the primary cell atlas dataset. The first column contains the Gene Omnibus ID of the arrays. The second and third column contains a description of the arrays and the array names and the last column the order of the arrays in the cluster plot (Fig. 1a) from left to right. Arrays stated as not selected were not depicted for the Fig. 1 but were used to produce together with 156 arrays to produce Figs. 5 and 7. (XLSX 39 kb) Additional file 4:
**Reconstructed models in SBML format.** The zipped file with 3 subfolders (cancer, monocyte and macrophage models, 156 primary cells models) contains 2 cancer models (cancer 1, cancer 2), 4 macrophage models (day 2, day 4, day 7, day 11), and 156 primary cells models, respectively. (7z 25 mb) Additional file 5: Table S5. Lists of the significantly up- (sheet 1) and down-regulated genes (sheet 2) (FDR < 0.05 and absolute (log fold change >1)) during monocyte to macrophage differentiation. Columns represent probe IDs, Gene symbol, log2 ratio between day 2 and day 11 of differentiation, fold change between the same time points, the p-value obtained after performing Empirical bayesian statistique (limma) and the FDR. (XLSX 186 kb) Additional file 6: Table S8. List of cofactor combinations that were not considered in the reaction equations for the determination of the entry points in Fig. 6. (XLSX 12 kb)
