# Supplementary material for: Integrated metabolic modelling reveals cell-type specific epigenetic control points of the macrophage metabolic network
Source: BMC Genomics. 2015 Oct 19;16:809. doi: 10.1186/s12864-015-1984-4 (PMC4617894; doi:10.1186/s12864-015-1984-4)
Supplement: Additional file 1: — FASTCORMICS allows fast context-specific metabolic model reconstruction using microarray data. This files furthermore contains: Table S1. Essentiality testing of different cancer models, Table S3. Hypergeometric test quantifying the enrichment of neoplasia related genes retrieved from DisGeNet, Table S6. Summary of the monocyte-macrophage models, Table S7. Confidence level of the included and excluded reactions of the monocytes macrophage models, as well as Figure S1. FASTCORMICS workflow, Figure S2. Correlation plot of predicted lactate secretion rates by context-specific cancer cell models and measured lactate secretion rates, Figure S3. Scatterplot of the fraction of active reactions in monocyte derived macrophages versus the fraction of active reactions in monocytes. (PDF 2196 kb) [file 12864_2015_1984_MOESM1_ESM.pdf]

Integrated metabolic modelling reveals cell-type specific epigenetic control points of the macrophage metabolic network

Maria Pires Pacheco, Elisabeth John, Tony Kaoma, Merja Heinäniemi, Nathalie Nicot, Laurent Vallar, Jean-Luc Bueb, Lasse Sinkkonen and Thomas Sauter

Additional Information

Additional text

FASTCORMICS allows fast context-specific metabolic model reconstruction using microarray data

The prospect of studying cell-type specific metabolism under numerous conditions or for example patient-specific metabolism in a diagnostic setting requires the capacity for fast creation of high-quality and robust metabolic models based on available data such as gene expression data. Recently we proposed an algorithm for the fast reconstruction of compact context-specific metabolic networks (FASTCORE) that reduces the reconstruction time of context-specific networks to the order of seconds[1]. In order to adapt FASTCORE for the integration of transcriptomics data from microarrays, we have developed a new workflow named FASTCORMICS (Additional Figure S1). As inputs FASTCORMICS requires microarray data and a GENRE of the organism of interest. Like FASTCORE, FASTCORMICS is devoid of arbitrary parameter settings and has a low computational demand with overall building times in the order of a few minutes. FASTCORMICS pre-processes microarray data with the discretization tool Barcode [2]. Barcode uses prior knowledge on the intensity distribution of each probe set for a given microarray platform to segregate between expressed and non-expressed genes. The preprocessing step with Barcode allows circumventing setting an arbitrary expression threshold to segregate between expressed and non-expressed genes, which is still commonly done [3–5]. As such a threshold is arbitrary and critical for the output metabolic models since due to this threshold complete branches, alternative pathways, or subsystems might be included or excluded, thereby significantly changing the functionalities of the model. Furthermore, Barcode shows a better correlation between predicted

expression and protein expression than competing discretization methods and decreases batch and lab-effects that affect measurements [2].

To validate FASTCORMICS we performed an essentiality assay on two generic cancer models that are based on Recon 1 and Recon 2 (cancer1 and cancer2, respectively) and generated by the FASTCORMICS workflow using existing microarray expression data from 59 cancer cell lines [6, 7]. The first model (cancer1) is composed of 810 reactions and is therefore bigger than the cancer model previously derived by Folger *et al*. (772 reactions) (Additional Table S1) [5]. The second model (cancer2) is composed of 1322 reactions. All reconstructed models are available in SBML format (Additional File S6). The assays performed on cancer1 and cancer2 predict 183 and 78 genes essential for cell growth, respectively (Additional Table S1). The predicted essential genes were compared to a list of 8000 genes ranked for essentiality by Luo *et al*. using a shRNA knockdown screen in several different cancer cell lines [8] to assess the predictive power of the FASTCORMICS models. In general, metabolic genes are slightly overrepresented in the top of the list as shown by Folger *et al* [5, 8], suggesting that metabolic genes are more essential than non-metabolic genes on average. As expected, the Recon 1 and Recon 2 models, even when further constrained by the medium composition (Additional Table S2, medium composition sheet), allowed identification of only a smaller set of essential genes and their distribution along the ranked list of essential genes was not significantly different from the distribution of all metabolic genes (Additional Table S1). Therefore, the predictive power of the reconstructed context-specific models is much better than either of the original GENREs. In contrast, the distribution of essential genes in the FASTCORMICS cancer models is different from the remaining metabolic genes and shifted towards the top of the ranked list as shown by a one-side

KS-test (p-value=0.0314 for cancer1 and p-value=0.0502 for cancer2), demonstrating that FASTCORMICS predictions are much more coherent with the experimental data. Moreover, comparison of the p-values to those obtained previously using the MBA algorithm (p-value=0.0284) [5, 9] suggests that FASTCORMICS performs with similar accuracy but with significantly lower running time (Additional Table S1) Consistently, a permutation test showed that the likelihood of finding a gene set of the same size with a better KS-score by chance is low (p-value=0.0063 for cancer1 and p-value=0.0351 for cancer2). In order to benchmark our workflow we also built cancer models using GIMME [3], iMAT [4] and mCADRE [10]. For GIMME and iMAT, the implementation of the Cobra toolbox [11] was run using as thresholds respectively the 75 and the 25 percentile for high and low expressed genes. For mCADRE the data was first discretized using Barcode [12] and then the implementation provided in the supplementary files of [10] was run. We also compared our workflow to PRIME[13]. PRIME uses microarray data and respective growth rate information to adapt the bounds of the input generic reconstruction. Thus it does not extract a context-specific sub-network from a general reconstruction and thereby differs from FASTCORMICS and the others algorithms discussed in this paper. Building a generic cancer model using PRIME was not possible as there is no generic growth rate. Instead the 32 models built by[14], were used to perform KO assays. 112 genes were essential in at least 90% of the 32 models (in fact these 112 genes were essential in all models). Out of the 112 genes, 81 were found in the ranked list of essential genes by [8] and used for p-value calculation.

We also tested iMat [4], but the algorithm does not guarantee that the biomass function is included in the model and therefore the knockout experiment could not be performed here.

In general, (Additional Table S1), more compact models, i.e. mCADRE cancer model, MBA cancer model, and cancer1 generated with FASTCORMICS, tend to predict a higher number of essential genes, respectively 169, 178 and 183, compared to models with a larger number of reaction, i.e. the GIMME cancer model that includes twice as many reactions as cancer1 and only 69 predicted essential genes. The aforementioned models also tend to perform better in the KO assay with the exception of mCADRE that identifies essential with a lower rank in the ranked essentiality list of Luo *et al* [8].

Context-specific models were built for the 59 cell lines integrating Recon1 and the cell line specific expression data with the FASTCORMICS workflow. The medium composition was used to constrain the inputs of the models (only input reactions for metabolites present in the medium were allowed to carry a flux). To obtain lactate secretion rates predictions in fmol/cell/h, the biomass coefficients were multiplied by 550 as described in[15]. Further, the bounds of the obtained models were multiplied with 1.5 to obtain a flux range consistent with the measured lactate rate. In order to guarantee lactate, glucose, oxygen and glutamine exchanges, the respective exchange reactions were added to the core set. To allow quantitative predictions for each context-specific models, the bounds of the inputs reactions of glucose and glutamine were fixed to match the experimental data. Additionally, the maximal uptake respectively production rate of alanine, serine, leucine, lysine, isoleucine, valine, arginine, threonine, tyrosine, phenylalanine, methionine, asparagine, choline, glycine, and tryptophan were constrained according to the experimental data. The uptake rates of cysteine, histidine, and myo-Inositol, which were not reported in the table, were set to zero. Random sampling was performed while optimizing for biomass production.

A solution could not be found for 7 cancer models, with these settings. For the other models a R2 value of 0.7 was obtained, indicating a good correlation of context specific predicted and measured lactate secretion rates.

As a second quality control step, a hypergeometric test showed that the neoplasia-associated genes retrieved from the DisGeNet database [16] are over-represented in the essential genes of both FASTCORMICS models (Additional Table S3). This indicates that FASTCORMICS can help to identify medically relevant genes. Further, among essential genes predicted in cancer1 and cancer 2 130 (71%) and 46 (59%) were known to be associated to cancer, respectively (DisGeNET [16], CCGD database[17]) or to be already predicted as essential by the generic model from which they were extracted.

Taken together, FASTCORMICS outperforms competing algorithms in speed and therefore allows generating robust high-quality models in a high-throughput manner. This will enable the use of metabolic modelling as a routine process for the analysis of large microarray data sets across different cell types and contexts.

Confidence levels of the reactions of the macrophage model

We compared the reactions of the macrophage model built with the FASTCORMICS workflow to a table (supplementary data 7) established by [18] that assigned confidence levels to the reactions of Recon1 in function of the evidence of expression in macrophage. 759 reactions of our model were found in the supplementary data 7 of [18], with 595 having a confidence level assigned. The remaining 410 reactions of our model not being listed in the Bordbar table [18] are due to a different annotation of Recon1 and Recon2 that was taken as input for our macrophage model. Of the 595

reactions with confidence information, 485 (82%) were assigned a high or medium confidence level by [18], 16 had a low and 94 are Exchanges/Transports added for modeling purposes, disassociations or spontaneous reactions to which no specific confidence level was assigned. No reactions were added that were shown not to be expressed in macrophages. Overall, this indicates a high confidence level for our reconstructed macrophage model.

Additional File S6: Reconstructed models in SBML format. The zipped file with 3 subfolders (cancer, monocyte and macrophage models, 156 primary cells models) contains 2 cancer models (cancer 1, cancer 2), 4 macrophage models (day 2, day 4, day 7, day 11), and 156 primary cells models, respectively**.**

1. Vlassis N, Pacheco MP, Sauter T: **Fast reconstruction of compact context-specific metabolic network models.** *PLoS Comput Biol* 2014, **10**:e1003424.

2. Zilliox MJ, Irizarry RA: **A gene expression bar code for microarray data**. *Nat Meth* 2007, **4**:911–913.

3. Becker SA, Palsson BØ: **Context-specific metabolic networks are consistent with experiments**. *PLoS Comput Biol* 2008, **4**:e1000082.

4. Zur H, Ruppin E, Shlomi T: **iMAT: an integrative metabolic analysis tool**. *Bioinformatics* 2010, **26**:3140–3142.

5. Folger O, Jerby L, Frezza C, Gottlieb E, Ruppin E, Shlomi T: **Predicting selective drug targets in cancer through metabolic networks**. *Mol Syst Biol* 2011, **7**.

6. Shankavaram UT, Varma S, Kane D, Sunshine M, Chary KK, Reinhold WC, Pommier Y, Weinstein JN: **CellMiner: a relational database and query tool for the NCI-60 cancer cell lines**. *BMC Genomics* 2009, **10**:277.

7. Pfister TD, Reinhold WC, Agama K, Gupta S, Khin SA, Kinders RJ, Parchment RE, Tomaszewski JE, Doroshow JH, Pommier Y: **Topoisomerase I levels in the NCI-60 cancer cell line panel determined by validated ELISA and microarray analysis and correlation with indenoisoquinoline sensitivity.** *Mol Cancer Ther* 2009, **8**:1878–1884.

8. Luo B, Cheung HW, Subramanian A, Sharifnia T, Okamoto M, Yang X, Hinkle G, Boehm JS, Beroukhim R, Weir BA, others: **Highly parallel identification of essential genes in cancer cells**. *Proc Natl Acad Sci* 2008, **105**:20380–20385.

9. Jerby L, Shlomi T, Ruppin E: **Computational Reconstruction of Tissue-specific Metabolic Models: Application to Human Liver Metabolism**. *Mol Syst Biol* 2010, **6;401**.

10. Wang Y, Eddy JA, Price ND: **Reconstruction of genome-scale metabolic models for 126 human tissues using {mCADRE}**. *BMC Syst Biol* 2012, **6**:153.

11. Schellenberger J, Que R, Fleming RMT, Thiele I, Orth JD, Feist AM, Zielinski DC, Bordbar A, Lewis NE, Rahmanian S, Kang J, Hyduke DR, Palsson BØ, R. , D: **Quantitative prediction of cellular metabolism with constraint-based models: the {COBRA} {T}oolbox v2.0.** *Nat Protoc* 2011, **6**:1290–1307.

12. McCall MN, Uppal K, Jaffee HA, Zilliox MJ, Irizarry RA: **The Gene Expression Barcode: leveraging public data repositories to begin cataloging the human and murine transcriptomes**. *Nucleic Acids Res* 2011, **39**(suppl 1):D1011–D1015.

13. Yizhak K, Gaude E, Le Dévédec S, Waldman YY, Stein GY, van de Water B, Frezza C, Ruppin E: **Phenotype-based cell-specific metabolic modeling reveals metabolic liabilities of cancer**. *Elife* 2014, **3**:1–23.

14. Yizhak K, Le Dévédec SE, Rogkoti VM, Baenke F, de Boer VC, Frezza C, Schulze A, van de Water B, Ruppin E: **A computational study of the Warburg effect identifies metabolic targets inhibiting cancer migration**. *Mol Syst Biol* 2014, **10,744**.

15. Gatto F, Miess H, Schulze A, Nielsen J: **Flux balance analysis predicts essential genes in clear cell renal cell carcinoma metabolism**. *Sci Rep* 2015, **5**:10738.

16. Queralt-Rosinach N, Furlong LI: **DisGeNET RDF: A Gene-Disease Association Linked Open Data Resource.** In *SWAT4LS*; 2013.

17. Starr T, Abbott K, Nyre E, Abrahante J, Ho Y-Y, Isaksson Vogel R: **Candidate Cancer Gene Database**.

18. Bordbar A, Lewis NE, Schellenberger J, Palsson BØ, Jamshidi N: **Insight into human alveolar macrophage and M. tuberculosis interactions via metabolic reconstructions**. *Mol Syst Biol* 2010, **6,422**.

19. Jain M, Nilsson R, Sharma S, Madhusudhan N, Kitami T, Souza AL, Kafri R, Kirschner MW, Clish CB, Mootha VK: **Metabolite Profiling Identifies a Key Role for Glycine in Rapid Cancer Cell Proliferation**. *Science* 2012, **336**:1040–1044.

Additional Table S1: Essentiality testing of different cancer models. Comparison of the number of essential genes found by an *in silico* essentiality assay to a ranked gene list established by Luo *et al*. based on the effect of shRNA knock-downs on the proliferation of cancer cells [8]. In Folger *et al*. [5] a gene is considered as essential if its knock-down results in a decrease of the growth rate of at least 1%. To allow for a comparison of the different methods the 1% criteria was applied here as well. *The number of essential genes was taken from Additional Table 3 Cancer Cytostatic Genes column KO Growth Rate (relative to WT) of [8].

Output model	Generic model	Contextualization method	Computational time	Size (reactions)	Essenti al genes	KS test p-value	Permutation p-value
Recon 1	Recon 1	None		2471	14	0.7623	0.7212
Recon2	Recon 2	None		5317	4	0.0231	0.0210
Medium constrained Recon 1 + biomass	Recon 1	Medium constrained		1922	78	0.1908	0.1444
Medium constrained Recon 2 + biomass	Recon 2	Medium constrained		4246	32	0.8260	0.7919
GIMME cancer model	Recon 1	GIMME	2497 sec	1749	69	0.0814	0.0465
PRIME cancer models (from [13])	Recon 1	PRIME		3788 (bounds are constrained)	112	0.0286	0.0152
mCADRE cancer model	Recon1	mCADRE	26356 sec	1037	169	0.1248	0.0228
MBA cancer model (from [9])	Recon 1	MBA	~2 hours /pruning step (1000 pruning steps)	772	178*	0.0284	0.0060
cancer1	Recon 1	FASTCORMICS	184 (preprocessing) +38 seconds	810	183	0.0314	0.0063
cancer2	Recon 2	FASTCORMICS	184 (preprocessing) + 288 seconds	1322	78	0.0502	0.0351

Additional Table S2: Medium composition and biomass formulation. The biomass equations are given with the metabolites abbreviations IDs as found in the models (sheet 2). In the sheet three, the 1st column contains the medium composition used to constrain Recon 1 using the metabolites abbreviation as used in the model. In the second column are displayed the medium composition for Recon 2.

Additional Table S3: Hypergeometric test quantifying the enrichment of neoplasia-related genes retrieved from DisGeNet [16], a database of disease-gene associations, in the set of essential genes of the different cancer models. In [5], a gene is considered essential if its knock-downs resulted in a decrease of the growth rate of at least 1%. To allow, a comparison with [5], the 1% criteria was applied as well.

Output model	Essential genes (EG)	EG in DisGeNet	Genes in the generic models (GG)	GG in DisGeNet	p-value
Recon1 (unconstrained)	14	6	1168	377	0.2792
Recon 2 (unconstrained)	4	1	1599	433	0.7176
Medium constrained Recon 1 + biomass	78	33	1168	377	0.0350
Medium constrained Recon 2 + biomass	32	14	1599	433	0.0299
GIMME cancer model	69	32	1168	377	0.0083
PRIME cancer models (from [14])	124	50	1496	449	4.69 e-4
mCADRE cancer model	169	73	1168	2635	2.474e-4
MBA cancer model (from [9])	178	84	1168	449	4.63e-6
cancer1	183	86	1168	377	4.28e-6
cancer2	106	45	1599	433	0.0295

Additional Table S4: List of selected 156 arrays ordered in function the Jaccard similarity index used for Figure 1 and of the remaining 745 arrays that compose the primary cell atlas dataset. The first column contains the Gene Omnibus ID of the arrays. The second and third column contains a description of the arrays and the array names and the last column the order of the arrays in the cluster plot (Figure 1a) from left to right. Arrays stated as not selected were not depicted for the figure 1 but were used to produce together with 156 arrays to produce Figure 5 and Figure 7.

Additional Table S5: Lists of the significantly up- (sheet 1) and down-regulated genes (sheet 2) (FDR<0.05 and absolute (log fold change ≥1)) during monocyte to macrophage differentiation. Columns represent probe IDs, Gene symbol, log2 ratio between day 2 and day 11 of differentiation, fold change between the same time points, the p-value obtained after performing Empirical bayesian statistique (limma) and the FDR.

Additional table S6: Summary of the monocyte-macrophage models

Model	reactions	metabolites	genes	Core reactions (including core transporters)	Inactive reactions
Day 2 model	978	858	614	462	806
Day 4 model	1055	918	594	605	759
Day 7 model	1202	1034	706	671	646
Day 11 model	1149	993	689	623	656

Additional Table S7: Confidence level of the included and excluded reactions of the monocytes macrophage models determined through the cross-validation step. Reactions with a high level of confidence are supported by at least two core reactions. Reactions with moderate confidence level are reactions only supported by barcode. Reactions with a weak confidence level are not supported by barcode, but needed to generate a consistent network model. Excluded reactions with a high confidence score were never included in any simulations suggesting the presence of other excluded reactions in the branch. Whereas, excluded reactions with a low confidence level were excluded only due to their low expression level.

Confidence level of					
**Model**	**Model reactions**			**Excluded reactions**	
	**high**	**moderate**	**weak**	**High**	**Weak**
Day 2 model	306	387	285	721	85
Day 4 model	391	491	173	667	96
Day 7 model	490	540	172	563	83
Day 11 model	441	507	201	593	63

Additional Table S8: List of cofactor combinations that were not considered in the reaction equations for the determination of the entry points in Figure 6.

Additional Figures

Additional Figure S1. FASTCORMICS workflow. Microarray data are discretized with Barcode in expressed (z-score > 5) and unexpressed genes (z-score < 0) that are mapped to the input model according to the Gene-Protein-Reactions rules. The FASTCORE core set is composed of reactions under the control of Barcode-supported genes. Optionally, the model can be constrained in function of the medium composition and a biomass function or the requirement to produce given metabolites can be added to the model. A modified version of FASTCORE, that allows the definition of a set of non-penalized reactions (in this study: Barcode-supported core reactions) is run. The modified version of FASTCORE forces the biomass function to carry a non-zero flux while penalizing the inclusion of non-core reactions. The output of the modified FASTCORE is then added to the core set and the modified FASTCORE is run again, now forcing all core reactions to carry a flux while penalizing non-core reactions. Transporters are removed from the core set, but are not penalized as explained in the main text. Finally a left-out cross-validation experiment can optionally be run to assign a confidence score to each reaction of the context-specific output model.

Additional Figure S2: Correlation plot of the predicted lactate secretion rates by context-specific cancer cell models and the lactate secretion rates measured by [19].

Additional Figure S3: A) Scatterplot of the fraction of active reactions in monocyte-derived macrophages versus the fraction of active reactions in monocytes. Each dot corresponds to a subsystem. 1: Biotin metabolism 2: Blood group synthesis 3:Dietary fiber binding 4: Thiamine metabolism 5: Ubiquinone synthesis 6:Vitamin D metabolism 7: Cytochrome metabolism 8: N-glycan synthesis 9: Histidine metabolism 10:Steroid metabolism 11:Transport, extracellular 12: Squalene and cholesterol synthesis 13:Vitamin C metabolism 14: Vitamin A metabolism 15: Galactose metabolism 16: Glycosphingolipid metabolism 17: Sphingolipid metabolism 18: Transport, mitochondrial 19: Transport, golgi apparatus 20: Tetrahydrobiopterin metabolism 21: Pyrimidine synthesis 22: Phenylalanine metabolism 23: Tyrosine metabolism 24: Bile acid synthesis 25: Keratan sulfate degradation 26:

Phosphatidylinositol phosphate metabolism 27: Transport, endoplasmic reticular 28: Tryptophan metabolism 29: Arginine and Proline Metabolism 30: Miscellaneous 31: Eicosanoid metabolism 32: Glycine, serine, alanine and threonine metabolism 33: Exchange/demand reaction 34 Lysine metabolism 35: O-glycan synthesis 36: Transport, nuclear 37: Keratan sulfate synthesis 38: Oxidative phosphorylation 39 Taurine and hypotaurine metabolism 40 Transport, peroxisomal 41: Triacylglycerol synthesis 42:Urea cycle 43: Vitamin B2 metabolism 44: Starch and sucrose metabolism 45: Transport, lysosome 46: Unassigned 47 Pyrimidine catabolism 48 Cholesterol metabolism 49: Glycerophospholipid metabolism 50: Propanoate metabolism 51: Alanine and aspartate metabolism 52: Fatty acid synthesis 53:Fructose and mannose metabolism 54: CoA catabolism 55: Methionine and cysteine metabolism 56: NAD metabolism 57: Pentose phosphate pathway 58: Vitamin B6 metabolism 59: Inositol phosphate metabolism 60 Purine catabolism 61 Valine, leucine, and isoleucine metabolism 62:Pyruvate metabolism 63: CoA synthesis 64: Glutathione metabolism 65: Aminosugar metabolism 66: Glyoxylate and dicarboxylate metabolism 67 Fatty acid oxidation 68 Nucleotide interconversion 69: Glutamate metabolism 70: Glycolysis/gluconeogenesis 71: beta-Alanine metabolism 72: Citric acid cycle 73: Folate metabolism 74: Purine synthesis 75: ROS detoxification 76:N-glycan degradation 77: C5-branched dibasic acid metabolism 78: Chondroitin sulfate degradation 79: Heme synthesis 80:Heparan sulfate degradation 81: Hyaluronan metabolism.

Additional Figure S4: *De novo* motif analysis tool MEME-ChIP was used to discover the enriched sequence motifs within the sequences underlying the reproducible identified active enhancer regions associated to upregulated genes in macrophages. The sequence logos of the enriched motifs corresponding to known TF binding sites in Jolma database and the associated TFs are listed below according to e-value. Only motifs with e-value $\leq$ 0.05 were considered.

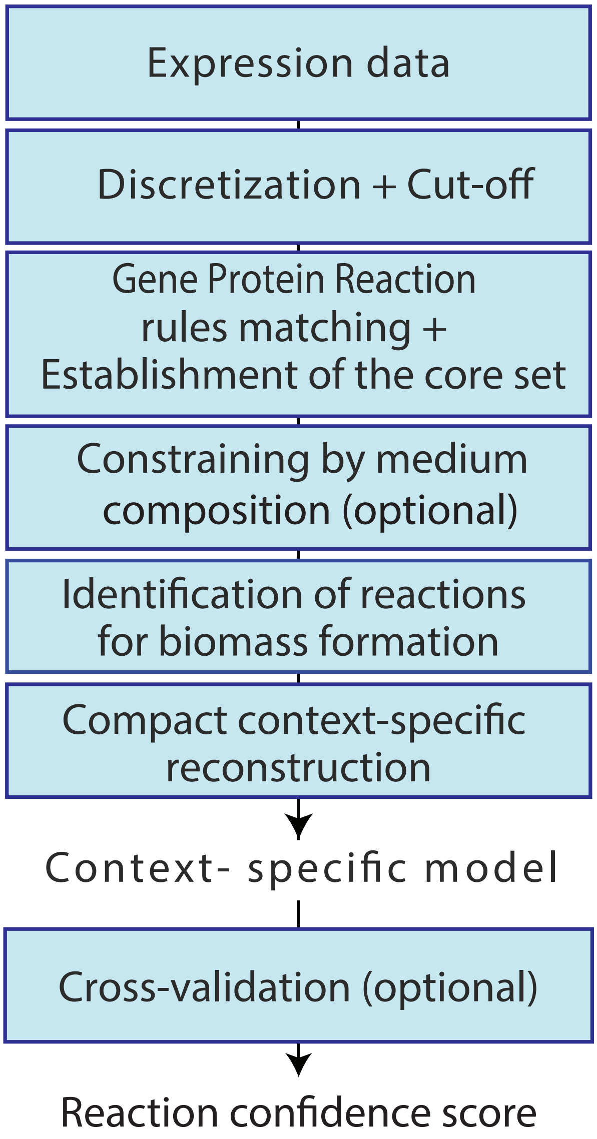

FASTCORMICS WORKFLOW

Additional Figure S1

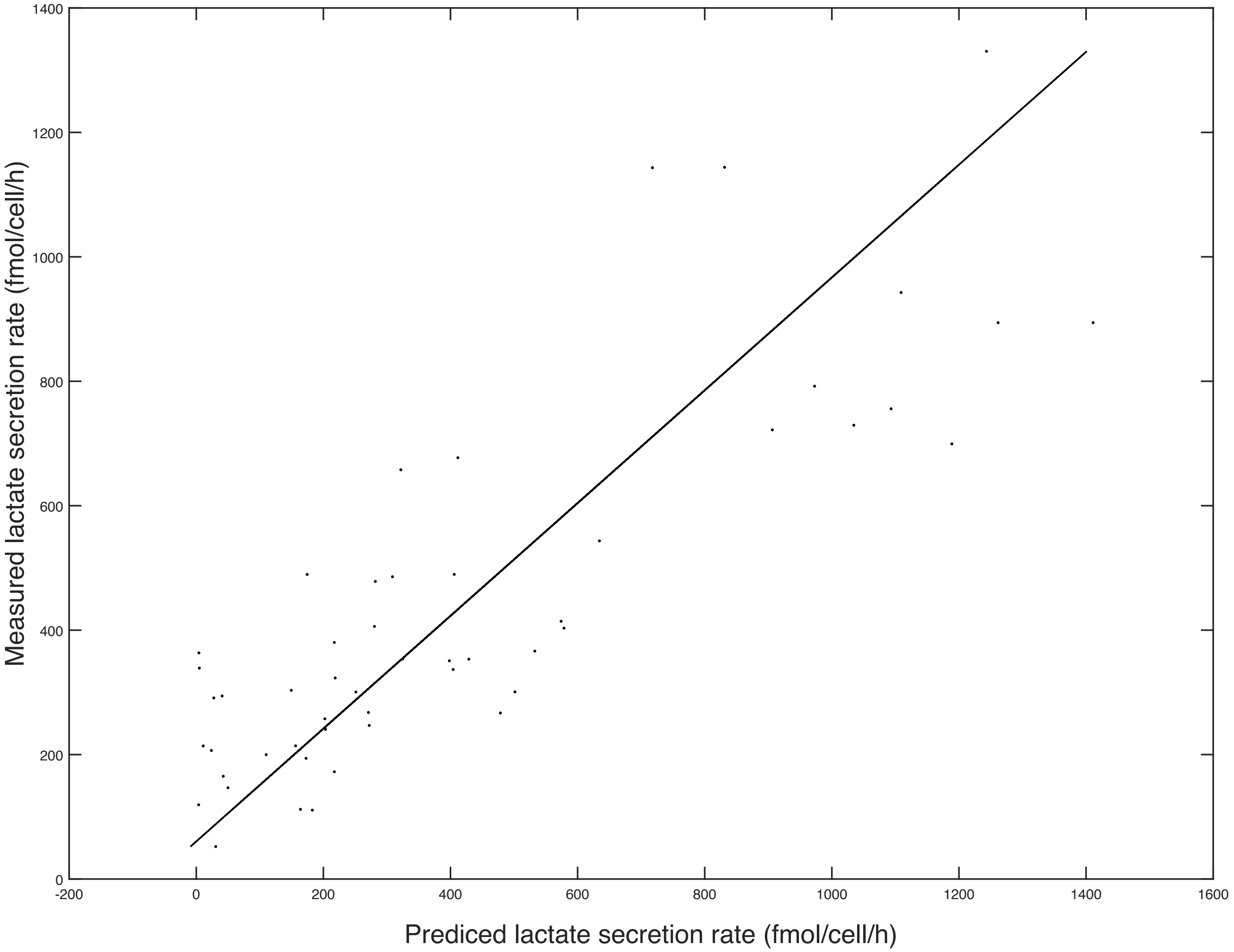

Additional Figure S2

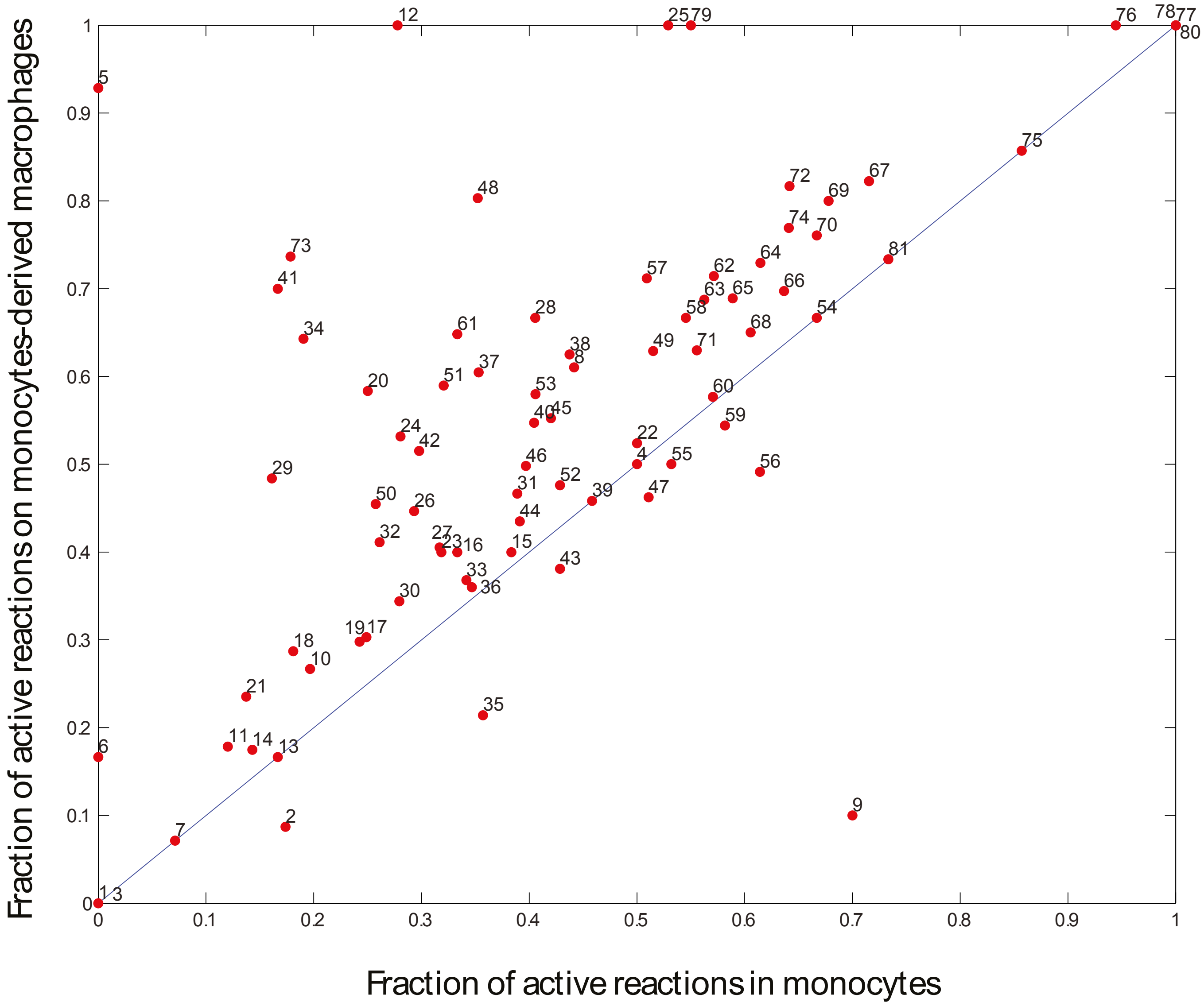

Additional Figure S3

No	Motif	e-value	TF	No	Motif	e-value	TF
1	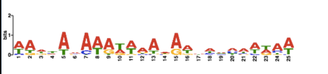	$3.9*10^{-45}$	CPEB1<br>FOXC1	15	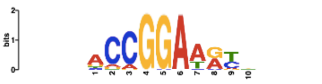	$2.0*10^{-3}$	ETS1
2	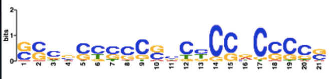	$7.9*10^{-12}$	Zfp740<br>ZNF740	16	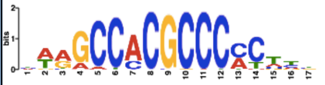	$2.4*10^{-3}$	SP4
3	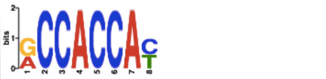	$3.1*10^{-6}$	GLI2	17	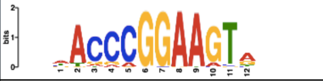	$2.6*10^{-3}$	ELF3
4	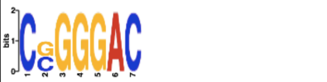	$1.3*10^{-5}$	SPDEF	18	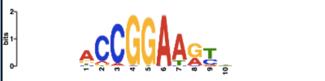	$3.1*10^{-3}$	ELK4
5	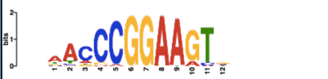	$2.7*10^{-5}$	ELF1	19	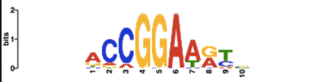	$3.2*10^{-3}$	FLI1
6	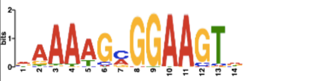	$6.9*10^{-5}$	SPIB	20	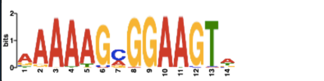	$4.4*10^{-3}$	SPI1
7	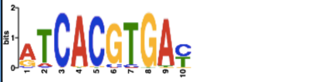	$8.5*10^{-5}$	SREBF2	21	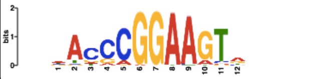	$6.2*10^{-3}$	EHF
8	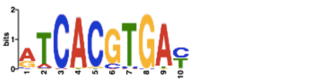	$8.8*10^{-5}$	Srebf1	22	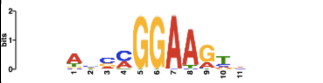	$6.4*10^{-3}$	ELF5
9	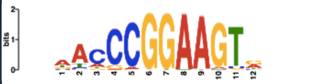	$1.2*10^{-4}$	ELF4	23	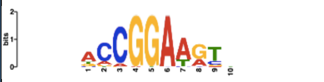	$6.4*10^{-3}$	FEV
10	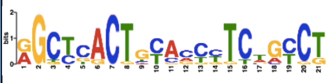	$3.7*10^{-4}$	SOX8	24	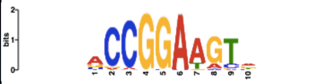	$7.0*10^{-3}$	GABPA
11	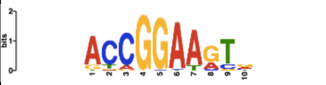	$4.7*10^{-4}$	ERF	25	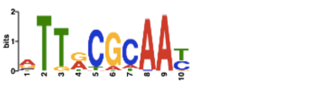	$1.1*10^{-2}$	CEBPD
12	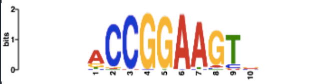	$5.9*10^{-4}$	ELK1	26	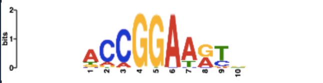	$1.1*10^{-2}$	ERG
13	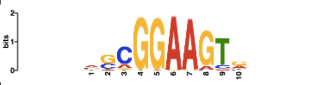	$9.5*10^{-4}$	ETV6	27	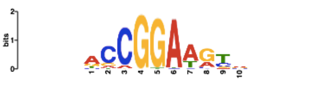	$2.9*10^{-2}$	ETV1
14	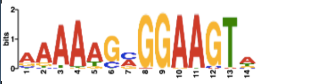	$1.4*10^{-3}$	Spic				

Additional Figure S4
